# Continuous warfarin administration versus heparin bridging therapy in post colorectal polypectomy haemorrhage: a study protocol for a multicentre randomised controlled trial (WHICH study)

**DOI:** 10.1186/s13063-020-04975-y

**Published:** 2021-01-07

**Authors:** Yasuaki Nagami, Taishi Sakai, Masafumi Yamamura, Masami Nakatani, Takayuki Katsuno, Takehisa Suekane, Hironori Uno, Hiroaki Minamino, Masatsugu Okuyama, Junichi Okamoto, Mitsutaka Kumamoto, Atsushi Noguchi, Kazuki Yamamori, Osamu Takaishi, Masahiro Ochi, Takako Miyazaki, Shigetsugu Tsuji, Hisatomo Ikehara, Koichiro Kawaguchi, Tomoyuki Hayashi, Tomohiko Mannami, Kazuki Kakimoto, Yoshihide Naito, Satoru Hashimoto, Zhaoliang Li, Yoriaki Komeda, Takaaki Kishino, Yoshinobu Yamamoto, Mikitaka Iguchi, Takuji Akamatsu, Toshiki Horii, Ko Miura, Takeshi Yamashina, Yuusaku Sugihara, Noboru Watanabe, Shu Kiyotoki, Ryoji Fujii, Masaki Murata, Satoshi Ono, Toshiaki Narasaka, Shinji Kitamura, Mitsuhiro Kono, Motohiko Kato, Hideto Kawaratani, Kyosuke Tanaka, Takao Yaoita, Shinjiro Yamaguchi, Keiichiro Abe, Takuji Kawamura, Yosuke Kinoshita, Kenichiro Imai, Haruka Fujinami, Tomoyuki Yada, Hayato Miyamoto, Hisako Yoshida, Yasuhiro Fujiwara

**Affiliations:** 1 Department of Gastroenterology, Osaka City University Graduate School of Medicine, 1-4-3, Asahimachi, Abeno-ku, Osaka, 545-8585 Japan; 2 Department of Gastroenterology, Baba Memorial Hospital, Osaka, Japan; 3 Department of Gastroenterology, Minamiosaka Hospital, Osaka, Japan; 4 Department of Gastroenterology, Izumiotsu Municipal Hospital, Osaka, Japan; 5 Department of Gastroenterology, Osaka City General Hospital, Osaka, Japan; 6 Department of Gastroenterology, Osaka Ekisaikai Hospital, Osaka, Japan; 7 Department of Gastroenterology, Ishikiriseiki Hospital, Osaka, Japan; 8 Department of Gastroenterology, Kashiwara Municipal Hospital, Osaka, Japan; 9 Department of Gastroenterology, Ikuwakai Memorial Hospital, Osaka, Japan; 10 Department of Gastroenterology, Nakae Hospital, Wakayama, Japan; 11 Department of Gastroenterology, Asakayama General Hospital, Osaka, Japan; 12 Department of Gastroenterology, Nagayoshi General Hospital, Osaka, Japan; 13 Department of Gastroenterology, Naniwa Ikuno Hospital, Osaka, Japan; 14 Department of Internal Medicine, Meijibashi Hospital, Osaka, Japan; 15 Center for Inflammatory Bowel Disease, Division of Internal Medicine, Hyogo College of Medicine, Hyogo, Japan; 16 Department of Gastroenterology, Ishikawa Prefectural Central Hospital, Ishikawa, Japan; 17 Department of Gastroenterology, Nihon University Hospital, Tokyo, Japan; 18 Division of Gastroenterology and Nephrology, Faculty of Medicine, Tottori University, Tottori, Japan; 19 Department of Gastroenterology, Kanazawa University, Ishikawa, Japan; 20 Department of Gastroenterology, Okayama Medical Center, Okayama, Japan; 21 Second Department of Internal Medicine, Osaka Medical Collage, Osaka, Japan; 22 Department of Gastroenterology and Hepatology, Fukui Prefectural Hospital, Fukui, Japan; 23 Division of Gastroenterology and Hepatology, Graduate School of Medical and Dental Sciences, Niigata University, Niigata, Japan; 24 Department of Gastroenterology, Takarazuka City Hospital, Hyogo, Japan; 25 Department of Gastroenterology and Hepatology, Kindai University Faculty of Medicine, Osaka, Japan; 26 Department of Gastroenterology and Hepatology, Center for Digestive and Liver Diseases, Nara City Hospital, Nara, Japan; 27 Department of Gastroenterological Oncology, Hyogo Cancer Center, Hyogo, Japan; 28 Second Department of Internal Medicine, Wakayama Medical University, Wakayama, Japan; 29 Department of Gastroenterology and Hepatology, Japanese Red Cross Society Wakayama Medical Center, Wakayama, Japan; 30 Department of Gastroenterology, Yuri Kumiai General Hospital, Akita, Japan; 31 Department of Gastroenterology, Tsuyama Chuo Hospital, Okayama, Japan; 32 Department of Gastroenterology and Hepatology, Osaka Red Cross Hospital, Osaka, Japan; 33 Department of Gastroenterology and Hepatology, Okayama University Hospital, Okayama, Japan; 34 Department of Gastroenterology, Graduate School of Medicine, Akita University, Akita, Japan; 35 Department of Gastroenterology, Shuto General Hospital, Yanai, Japan; 36 Department of Gastroenterology, Tonan Hospital, Hokkaido, Japan; 37 Department of Gastroenterology, Shiga University of Medical Science, Shiga, Japan; 38 Department of Gastroenterology, Chiba-Nishi General Hospital, Chiba, Japan; 39 Department of Gastroenterology, University of Tsukuba, Ibaraki, Japan; 40 Department of Gastroenterology and Oncology, Institute of Biomedical Sciences, Tokushima University Graduate School, Tokushima, Japan; 41 Department of Gastrointestinal Oncology, Osaka International Cancer Institute, Osaka, Japan; 42 Division of Gastroenterology and Hepatology, Department of Internal Medicine, Keio University School of Medicine, Tokyo, Japan; 43 Division of Research and Development for Minimally Invasive Treatment, Cancer Center, Keio University School of Medicine, Tokyo, Japan; 44 Department of Gastroenterology, Nara Medical University, Nara, Japan; 45 Department of Endoscopy, Mie University Hospital, Mie, Japan; 46 Department of Gastroenterology, Yamagata University Faculty of Medicine, Yamagata, Japan; 47 Division of Gastroenterology, Kansai Rosai Hospital, Hyogo, Japan; 48 Department of Gastroenterology, Dokkyo Medical University, Tochigi, Japan; 49 Department of Gastroenterology, Kyoto Second Red Cross Hospital, Kyoto, Japan; 50 Division of Endoscopy, Shizuoka Cancer Centre, Shizuoka, Japan; 51 Department of Gastroenterology, Toyama University Hospital, Toyama, Japan; 52 Division of Gastroenterology & Hepatology, Kohnodai Hospital, National Center for Global Health and Medicine, Chiba, Japan; 53 Department of Gastroenterology, Hanwa Sumiyoshi General Hospital, Osaka, Japan; 54 Department of Medical Statistics, Osaka City University Graduate School of Medicine, Osaka, Japan

**Keywords:** Colorectal polypectomy, Anticoagulants, Warfarin, Heparin bridge, Vitamin K antagonist

## Abstract

**Background:**

Endoscopic removal of colorectal adenoma is considered an effective treatment for reducing the mortality rates associated with colorectal cancer. Warfarin, a type of anticoagulant, is widely used for the treatment and prevention of thromboembolism; however, bleeding may increase with its administration after polypectomy. In recent times, a high incidence of bleeding after endoscopic polypectomy has been reported in patients receiving heparin bridge therapy. However, previous studies have not compared the bleeding rate after endoscopic colorectal polypectomy between patients who continued with anticoagulant therapy and those who received heparin bridge therapy. We hypothesised that endoscopic colorectal polypectomy under the novel treatment with continuous warfarin is not inferior to endoscopic colorectal polypectomy under standard treatment with heparin bridge therapy with respect to the rate of postoperative bleeding. This study aims to compare the efficacy of endoscopic colorectal polypectomy with continuous warfarin administration and endoscopic colorectal polypectomy with heparin bridge therapy with respect to the rate of postoperative bleeding.

**Methods:**

We will conduct a prospective multicentre randomised controlled non-inferiority trial of two parallel groups. We will compare patients scheduled to undergo colorectal polypectomy under anticoagulant therapy with warfarin. There will be 2 groups, namely, a standard treatment group (heparin bridge therapy) and the experimental treatment group (continued anticoagulant therapy). The primary outcome measure is the rate of postoperative bleeding. On the contrary, the secondary outcomes include the rate of cumulative bleeding, rate of overt haemorrhage (that does not qualify for the definition of haemorrhage after endoscopic polypectomy), incidence of haemorrhage requiring haemostasis during endoscopic polypectomy, intraoperative bleeding during endoscopic colorectal polypectomy requiring angiography, abdominal surgery and/or blood transfusion, total rate of bleeding, risk factors for postoperative bleeding, length of hospital stay, incidence of thromboembolism, prothrombin time-international ratio (PT-INR) 28 days after the surgery, and incidence of serious adverse events.

**Discussion:**

The results of this randomised controlled trial will provide valuable information for the standardisation of management of anticoagulants in patients scheduled to undergo colorectal polypectomy.

**Trial registration:**

UMIN-CTR UMIN000023720. Registered on 22 August 2016

## Background

Colorectal cancer is the third most common cancer and the fourth-largest cause of cancer-related mortality [1]. Endoscopic removal of colorectal adenoma, a precursor of colorectal cancer, is considered an effective treatment for reducing the mortality associated with colorectal cancer [2–4]. Endoscopic resection is widely acknowledged as the standard treatment for colorectal tumours because of its technical simplicity and lower rate of adverse events [5, 6]. Endoscopic resection of colorectal polyp results in postoperative bleeding in 0.9–7% of the cases; however, the rate is reported to increase by approximately 10% in patients taking anticoagulants [7]. A vitamin K antagonist and a type of anticoagulant, warfarin, is widely used for the treatment and prevention of thromboembolism (venous thrombosis, myocardial infarction, cerebral embolism, and thrombosis, among others). Discontinuation of anticoagulants increases the risk of thrombosis to approximately 3% of the patients [8]. Therefore, the guidelines for gastroenterological endoscopy in patients undergoing antithrombotic treatments, issued by the American Society for Gastrointestinal Endoscopy (ASGE) and the Japanese Society of Gastroenterological Endoscopy (JGES), recommend that patients discontinue anticoagulants and replace them with heparin before undergoing endoscopic procedures. This recommendation is based on the results of a case series of patients who received heparin bridge therapy [9, 10]. However, a high incidence of bleeding after endoscopic polypectomy has been reported in patients receiving heparin bridge therapy in recent studies [11, 12].

In contrast, in cases of atrial fibrillation wherein the patients discontinued warfarin without heparin bridge therapy during the elective invasive procedure, the incidence of thromboembolism was lower than that noted for the heparin bridge therapy group, and replacement of heparin increased the rate of haemorrhage [13]. Patients who were continued on warfarin during implantation of pacemakers/defibrillators were shown to have a significantly lower risk of postsurgical haemorrhage and haematoma than patients on heparin bridge therapy. The two groups did not show any difference in the risk of embolism [14, 15].

Thus, we hypothesised that colorectal polypectomy under continued anticoagulation therapy may be associated with a lower rate of bleeding rate than colorectal polypectomy under heparin bridge therapy and that continuing warfarin is not inferior to heparin bridge therapy. Previous studies have not compared the rate of postoperative bleeding after endoscopic colorectal polypectomy between patients who continued anticoagulants and those who received heparin bridge therapy. If the rate of bleeding after endoscopic polypectomy in patients who continued with warfarin is not lower than that for those receiving heparin bridge therapy, there is no need for long-term hospitalisation, and the associated cost and patient burden associated with heparin bridge therapy will decrease. Endoscopic colorectal polypectomy can be performed more safely without concerns about the increased risk of thrombosis associated with the discontinuation of warfarin.

This study was designed with the aim to prove that endoscopic colorectal polypectomy under the novel treatment of continuous warfarin is not inferior to the same procedure under the standard treatment of heparin bridge, in terms of the postoperative bleeding rate.

## Methods and design

### Study design

**Fig. 1:**
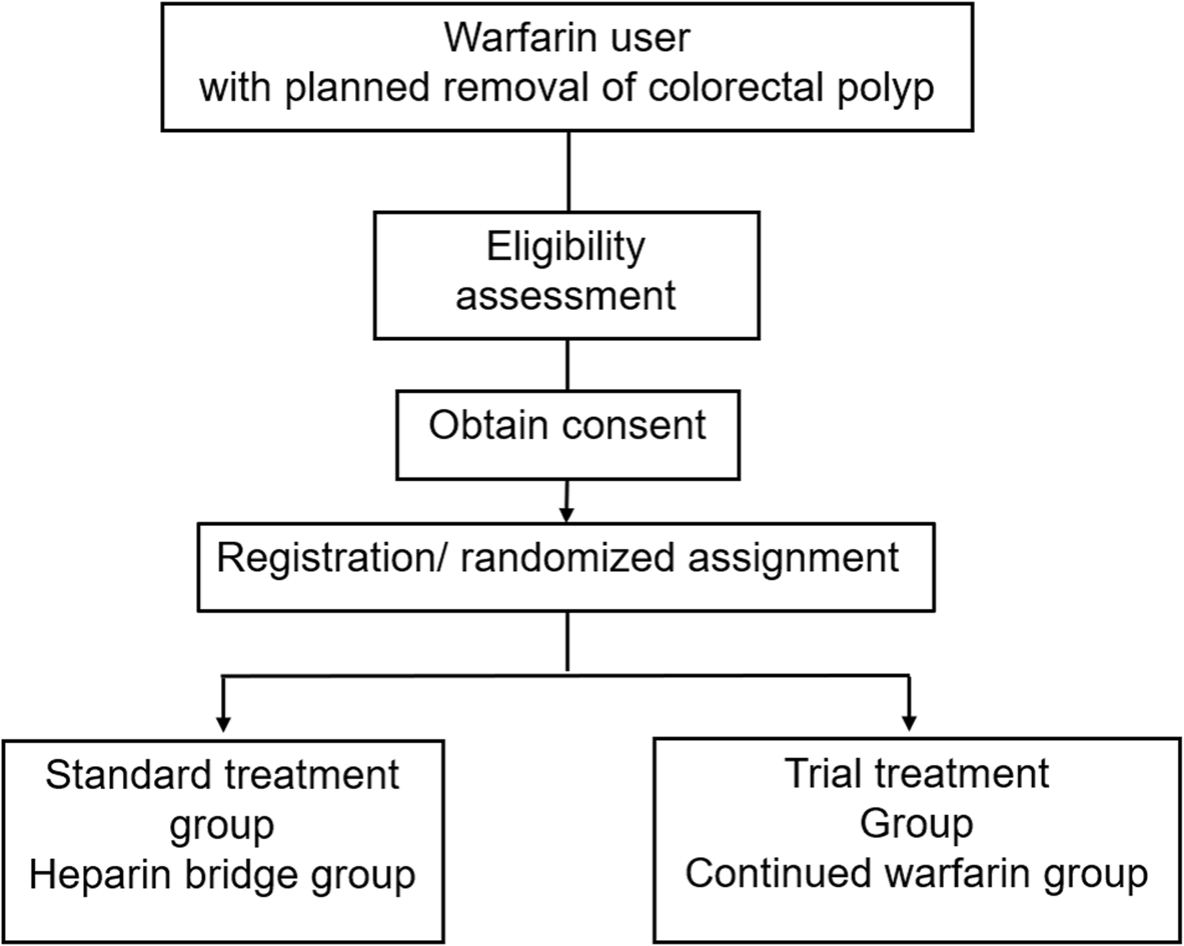
Diagram of the study design

This trial is a prospective multicentre randomised controlled (RCT) non-inferiority trial of parallel two-group (Fig. 1). We compare the incidence of postoperative bleeding associated with colorectal polypectomy of patients under anticoagulant therapy of warfarin between the standard treatment group (heparin bridging therapy) and experimental treatment (continued anticoagulant therapy). This is a multicentre trial that is conducted at Osaka City University Hospital and 51 Japanese hospitals.

### Approvals

It is conducted according to the ethical guidelines for clinical studies while considering the patients’ human rights and privacy. The protocol of this study was approved by the Institutional Review Board (IRB) of the Osaka City University Hospital (No. 3432) and the IRB of each participating institution, and it has also been registered at UMIN-CTR (UMIN000023720).

### Study patients

Subjects of this study include patients who undergo consultation at institutions participating in this study, who are taking warfarin in an outpatient or inpatient setting, and who are planned to undergo colorectal polypectomy, the target disease of this study.

For patients who provide written consent after receiving study explanations, we will confirm that they satisfy the inclusion criteria and that they do not meet any of the exclusion criteria.

#### Inclusion criteria

Patients who satisfy the following criteria are included:

1. Patients with polyps that can undergo en-bloc resection of the large intestine (cecum, colon, and rectum) and who are scheduled for endoscopic colorectal polypectomy
2. Patients who have been taking warfarin for at least 2 weeks prior to the day of endoscopic colorectal polypectomy
3. Patients who are at least 20 years old at the time of obtaining consent
4. A written consent is provided based on the patient’s free will, after he or she has a thorough understanding of the instructions given regarding study participation

#### Exclusion criteria

1. Patients with a history of enrolment in this study
2. Patients with inflammatory bowel disease, familial adenomatous polyposis, and Peutz-Jeghers syndrome
3. Patients whose clinical course cannot be followed up to 28 days after treatment
4. Patients with either a history of bleeding with blood transfusion of 2 red blood cell (RBC) units or more, haemoglobin (Hb) reduction of ≥ 2 g/dL, or haemostasis treatment within 6 weeks before polypectomy
5. Dialysis patients
6. Patients whose blood test showed a platelet count of less than 50,000/μL within 12 weeks before polypectomy
7. Patients with coagulation dysfunctions
8. Pregnant patients
9. Lactating patients
10. Patients who are allergic to heparin and/or warfarin
11. Other cases determined to be unfit for study by an investigator

### Trial intervention

**Fig. 2:**
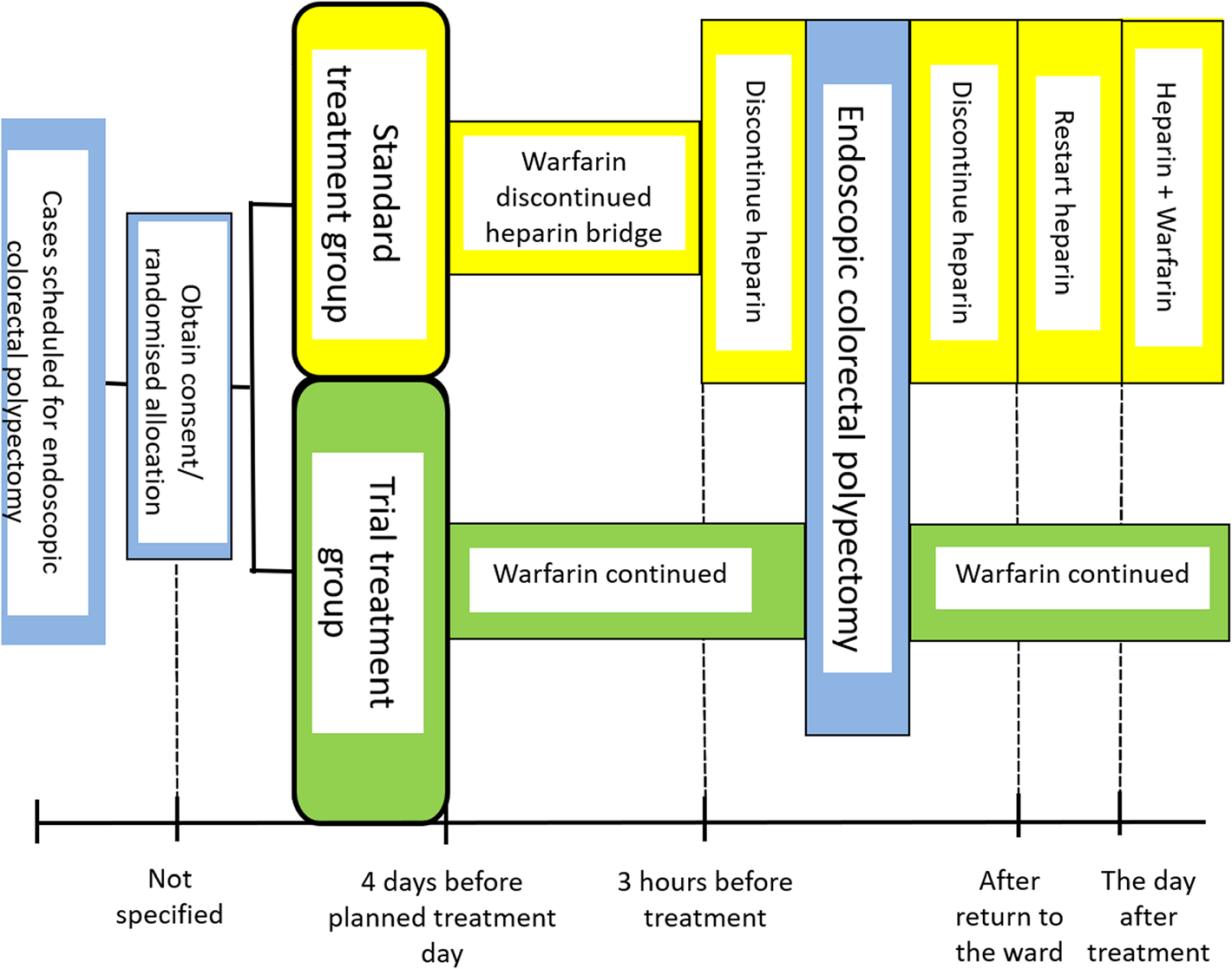
Study outline

Enrolled patients under anticoagulant therapy of warfarin will be randomised to undergo polypectomy for colorectal polyps under the standard treatment (heparin bridging therapy) or experimental treatment (continued anticoagulant therapy) (Fig. 2, Table 1).

**Table 1:**
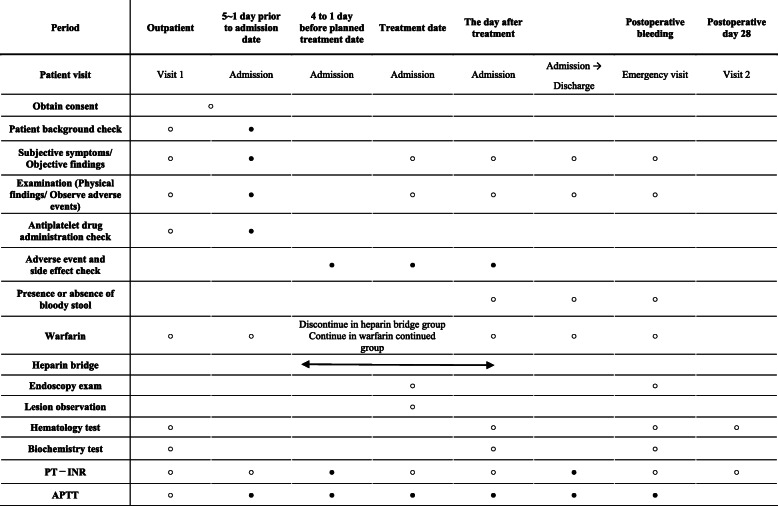
Schedule of this study

Consent can be obtained in the outpatient setting as well. In addition, a patient can be treated if the PT-INR becomes 3 or less after hospitalisation. If a blood test cannot be done on postoperative day 28, it can be done up to postoperative day 35

^○^Items that must be implemented

^●^Items that will be implemented as necessary

#### Standard treatment group (heparin bridge group)

Discontinue warfarin from 4 days prior to the procedure date, and start heparin bridge. For heparin bridge, continuous intravenous infusion of unfractionated heparin of 10,000 to 20,000 units will be given per day at a dose of approximately 200 U/kg/day. The dose will be controlled to keep the activated partial thromboplastin time (APTT) within 1.5–2.5 times the control. Measure APTT/the prothrombin time-international ratio (PT-INR) in the morning of the treatment day and confirm that PT-INR is less than 1.5. The day of endoscopic treatment will be postponed if the PT-INR is 1.5 or more on the scheduled date. Heparin bridge should be discontinued 3 h before endoscopic treatment and is restarted as soon as the patient returns to the ward from the endoscopic treatment. If there is no sign of bleeding as indicated above by the next morning, resume the same amount of warfarin that the patient received before discontinuation. After that, confirm that PT-INR has reached the therapeutic range (1.5 or more), and discontinue heparin.

#### Trial treatment group (continued warfarin group)

Patients will continue on outpatient warfarin dose. Measure APTT/PT in the morning of the endoscopic treatment day and confirm that PT-INR is 3 or below. The treatment day will be postponed if PT-INR is > 3 on the scheduled date. Continue warfarin on the day of treatment as well with the outpatient dose.

### Endoscopic procedures

Follow the standard bowel preparation and sedation during the endoscopic procedure at each facility’s regular medical practice of each institution. We will take appropriate measures to allow this study to take place under the best possible conditions. For endoscopic colorectal polypectomy including endoscopic mucosal resection (EMR) in this study, the procedure will be performed by, or under the guidance of, a specialist physician qualified by the Japan Gastroenterological Endoscopy Society. Submucosal injection can be performed, and the type of injection will not be specified. It will be completed following a regular clinical practice. The electric current setting and the type of snare used will not be specified. Each facility will follow its regular clinical practice and both bipolar and monopolar snares may be used, but not perform cold polypectomy. After polypectomy, if an active bleeding without spontaneous haemostasis or exposed blood vessels is observed, additional haemostasis techniques such as clipping or ablation should be performed. After polypectomy, the resection site will be prophylactically clipped as a general rule in all cases. Nothing per os on the day of the treatment and any fluid replacement will be determined by each investigator. Resume meals if there is no sign of bleeding such as bloody stools, decreasing Hb level, and fluctuating vital signs.

### Outcome measures

#### Primary outcome measure

Postoperative bleeding rate that the number of cases with postoperative bleeding/the number of cases that underwent polypectomy. Postoperative bleeding is defined when bleeding is observed with one or more of the following within 28 days after polypectomy:

1. Bloody stool with an Hb decrease of 2 g/dL or more
2. Overt bloody stool treated with endoscopic haemostasis, angiography, surgery, and/or blood transfusion.

Emergency colonoscopy will be performed in the following cases: twice or more of persistent bloody stool, bloody stool with changes in vital signs (systolic blood pressure < 100 mmHg or pulse > 90 beats/min), or bloody stool with an Hb decrease of 2 g/dL or more.

#### Secondary outcome measures

1. Cumulative bleeding rate
2. Rate of overt haemorrhage that does not satisfy the definition of haemorrhage after endoscopic polypectomy
3. Incidence rate of haemorrhage that required haemostasis during endoscopic polypectomy
4. Cases that required haemostasis during the polypectomy: cases where a haemostasis technique such as clipping was performed for bleeding without spontaneous haemostasis
5. Intraoperative bleeding during endoscopic colorectal polypectomy requiring angiography, surgery, and/or blood transfusion
6. Total bleeding rate (postoperative bleeding + above 2 + 3)
7. Risk factors for postoperative bleeding
8. Number of hospitalisation days
9. Incidence rate of thromboembolism
10. PT-INR 28 days after the polypectomy (if it is difficult to perform a blood test on postoperative day 28, it can be performed up to postoperative day 35)
11. Rate of serious adverse events

### Randomisation

In this study, the enrolled cases will undergo dynamic randomisation using the online registration allocation system at the data centre within the Osaka City University Hospital Center for Clinical Research and Innovation. This study will use the dynamic randomised allocation by minimisation in order to control for the number of colorectal polyps between the two groups and the background risk factors of bleeding. The following three allocation adjustment factors will be used in the minimisation technique: (1) institutions, (2) the number of polyps known in advance, and (3) concomitant use of antiplatelet drugs (or lack thereof). The researchers at participating institutions will not be informed of the detailed procedure of the randomised allocation method.

### Blinding

This study will not be blinded.

### Sample size

Based on the post-procedural bleeding rate after endoscopic polypectomy in a previous report, we assume the post-procedural bleeding rate of 14% for warfarin continued cases, and 20% for heparin bridged cases. Non-inferiority margin was set to 5%. Given *α* value of 0.05 and power of 0.8, we considered an enrolment of about 144 cases in each group to be appropriate. The target number of cases was set at 158 in each group, for a total of 316, assuming that a little less than 10% of all cases may be discontinued or are ineligible.

### Statistical analysis

The target analysis group will be set as the full analysis set (FAS), defined as subjects who were assigned to this study, who took the study drug at least once and were evaluated for efficacy at least once after study drug administration. Further analysis of per protocol set (PPS) that meets the study protocol will also be done complementarily.

The primary assessment parameter of postoperative bleeding rate between groups will be analysed using a generalised linear model with groups as fixed effects adjusted for allocation factors. We test the null hypothesis that the risk ratios of both groups are equal (= 1). The significance level is 5% (one-sided), and the 95% confidence interval will be calculated. The trial treatment will be considered noninferior to standard treatment if 95% confidence interval of risk ratio does not exceed 1.05.

The secondary assessment parameter in item 1 will be analysed by the same method as the primary endpoint. If we can show non-inferiority in each endpoint, we would also like to analyse the superiority of each endpoint. In 1 and 2, a Kaplan-Meier curve of cumulative incidence for each group will be created and its 95% confidence interval will be calculated. The confidence interval will be calculated for the double logarithmic transformation value. In 3, 4, 5, 8, and 10 parameters, the Kaplan-Meier curve of cumulative incidence for each group will be created and its 95% confidence interval will be calculated. The confidence interval will be calculated for the double logarithmic transformation value. The log rank test will be performed for comparison between groups (superiority). In parameter 6, in order to identify risk factors, the Cox regression model adjusted for all allocation factors will be performed and estimate the hazard ratio and 95% confidence interval.

### Interim analyses

To examine whether the study can be continued or not, an interim analysis will be conducted 28 days after the registration of 100 cases. The main purpose of the interim analyses will be to confirm the safety of the protocol’s treatment. In this case, only serious adverse events are evaluated and not primary assessment parameters.

### Data registration

Data will be entered into web-based Electronic Data Capture (EDC) system at the data centre within the Osaka City University Hospital Center for Clinical Research and Innovation by trial investigators or site investigators. The trial database will be created from the EDC system.

## Discussion

Since anticoagulant therapy increases the risk of postoperative bleeding after colorectal polypectomy, it is important to manage anticoagulant therapy before, during, and after the procedure; however, there is no consensus about the appropriate approach. An RCT showed that interruption of warfarin could reduce bleeding and that the incidence of thromboembolism was less than that with heparin bridging therapy during an elective invasive procedure [13]. However, most procedures in the study were low-risk procedures like conventional endoscopy with biopsy, which has a low risk of bleeding and thromboembolism. Moreover, only patients with atrial fibrillation and a low risk of thromboembolism and bleeding were enrolled, resulting in a low rate of bleeding of 3.2%, even in the group on heparin bridge therapy. A high incidence of postoperative bleeding of 20.0% has been reported with the standard treatment using heparin bridge therapy in cases of colorectal polypectomy [11]. Another study reported that the post-polypectomy rate of bleeding in patients who continued on warfarin therapy was 14% [16]. During implantation of pacemakers/defibrillators, continued warfarin therapy reduced the incidence of haemorrhage and haematoma, compared to heparin bridge therapy, from 16 to 3.5% [14]. It was also reported that the rate of bleeding was lower in the group that continued anticoagulant therapy during endoscopic submucosal dissection of the stomach [17]. There is no comparative study about the rate of postoperative bleeding after endoscopic colorectal polypectomy between patients who continued on anticoagulant therapy and those who received heparin bridging therapy. Hence, there is a need for a multicentre RCT to determine the benefits and risks of continued warfarin therapy during polypectomy for colorectal polyps.

Detailed clinical outcomes including adverse events of colorectal polypectomy in patients who continued warfarin therapy are unknown. We will examine the rate of cumulative bleeding, minor bleeding, haemorrhage during the procedure, total rate of bleeding, and serious adverse events between the two groups as secondary endpoints. Although this study is a non-inferiority study, we also aim to determine whether warfarin administration or heparin bridge therapy might be a risk factor for postoperative haemorrhage and whether there are other risk factors for postoperative haemorrhage. In addition, we plan to determine whether the incidence of thromboembolism in patients on heparin bridge therapy patients differs from that in patients continuing warfarin. Heparin bridge therapy requires a 24-h continuous drip infusion. Hence, it is an extra burden on the patients and medical staff, and longer duration of hospitalisation is necessary due to additional time required while switching from warfarin to heparin before and after treatment. There is also a corresponding increase in the medical costs. A previous study showed that a median of 14 (8–37) days of hospitalisation was necessary in patients undergoing heparin bridge therapy for the colorectal polypectomy procedure [11]. We will compare the duration of hospitalisation as a secondary endpoint between the two groups.

The limitations of this study include the non-blinded design and the exclusion of novel anticoagulants and direct oral anticoagulants (DOAC) since the rate of postoperative bleeding after endoscopic colorectal polypectomy has not been clarified.

The safer and more cost-effective techniques for the treatment of colorectal polyps could be promising for patients on anticoagulants. The results of this RCT would provide valuable information for future standardisation of the management of anticoagulant therapy in patients who are scheduled to undergo colorectal polypectomy.

## Trial status

First version protocol was approved on 4 August 2016. This protocol is the 7th version which was approved on 9 June 2020. Amendments mainly include adding cooperative institutions and researchers, because it was difficult to recruit participants in earlier period due to decreasing patients with warfarin.

Recruitment was begun since 13 October 2016. Participant recruitment is in progress. Recruitment is expected to end by August 2022.

## Supplementary Information

**Additional file 1.** Protocol of WHICH study ver. 7.

**Additional file 2.** Statistical analysis plan.

## Data Availability

Access to the dataset will be limited to the data centre members.

## Abbreviations

RCT: Randomised controlled
ASGE: American Society for Gastrointestinal Endoscopy
JGES: Japanese Society of Gastroenterological Endoscopy
IRB: Institutional Review Board
RBC: Red blood cell
Hb: Haemoglobin
APTT: Activated partial thromboplastin time
PT-INR: Prothrombin time-international ratio
EMR: Endoscopic mucosal resection
FAS: Full analysis set
PPS: Per protocol set
