# Supplementary material for: Continuous warfarin administration versus heparin bridging therapy in post colorectal polypectomy haemorrhage: a study protocol for a multicentre randomised controlled trial (WHICH study)
Source: Trials. 2021 Jan 7;22:33. doi: 10.1186/s13063-020-04975-y (PMC7791998; doi:10.1186/s13063-020-04975-y)
Supplement: Supplementary file 1 — Additional file 1. Protocol of WHICH study ver. 7. [file 13063_2020_4975_MOESM1_ESM.docx]

The Japanese Gastroenterological Association　2018　Multicenter Joint Study Grant

**Medical Research Protocol**

Continuous Warfarin administration versus Heparin bridging therapy in post colorectal polypectomy hemorrhage

: A randomized controlled multicenter study

Short title: WHICH study

The clinical trial registry website and the clinical trial number

http://www.umin.ac.jp (number UMIN000023720)

Principal investigator Yasuaki Nagami

Department of Gastroenterology, Osaka City University Graduate School of Medicine

1-4-3, Asahimachi, Abeno-ku, Osaka 545-8585, Japan

Tel: +81-6-6645-3811

ver.1.0 April 28, 2016

ver.2.0 June 29, 2017

ver.3.0 October 31, 2017

ver.4.0 January 31, 2018

ver.5.0 April 30, 2018

ver.6.0 August 31, 2018

ver.7.0 June 9, 2020

# Overview

## Scheme

Warfarin user

With planned removal of colorectal polyp

Eligibility assessment

Obtain consent

Registration/randomised assignment

Trial treatment group

Continued warfarin group

Standard treatment group

Heparin bridge group

## Purpose

Compared with conventional method where patients on warfarin potassium (warfarin) switch to heparin sodium (heparin) for endoscopic colorectal polypectomy, this study was designed to demonstrate that the trial treatment method of endoscopic colorectal polypectomy under continuous warfarin is not inferior to the conventional heparin bridge method in terms of the frequency of postoperative bleeding events.

## Subjects

Subjects of this study include patients who underwent consultation at institutions participating in this study, who are taking warfarin in an outpatient or inpatient setting, and who are planned to undergo colorectal polypectomy, the target disease of this study.

**・Inclusion Criteria**

Patients who satisfy the following criteria are included.

1. Patients with polyps that can undergo en-bloc resection of the large intestine (cecum, colon, and rectum), and who are scheduled for endoscopic colorectal polypectomy
2. Patients who have been taking warfarin for at least 2 weeks prior to the day of endoscopic colorectal polypectomy
3. Patient who are at least 20 years old at the time of obtaining consent

4) A written consent is provided based on the patient’s free will, after he or she has a thorough understanding of the instructions given regarding study participation

[Criteria rationale]

1) and 2) Targets are patients who continue to take anticoagulants and undergo endoscopic colorectal polypectomy

3) and 4) For safety considerations

Note 1) Patients taking direct oral anticoagulant drugs (DOAC) are not included.

Note 2) Included also are cases that are diagnosed as resectable en-bloc, but actually undergo piecemeal resection.

　　Exclusion Criteria

Patients who meet any of the following criteria are excluded.

1. Patients with a history of enrolment in this study
2. Patients with inflammatory bowel disease, familial adenomatous polyposis, and Peutz-Jeghers syndrome
3. Patients whose clinical course cannot be followed up to 28 days after treatment
4. Patients with either a history of bleeding with blood transfusion of 2 RBC units or more, Hb reduction of ≧ 2g/dL, or haemostasis treatment within 6 weeks before surgery
5. Dialysis patients
6. Patients whose blood test showed a platelet count of less than 50,000/μL within 12 weeks before surgery
7. Patients with coagulation dysfunctions
8. Pregnant patients
9. Lactating patients
10. Patients who are allergic to heparin and/or warfarin
11. Other cases determined to be unfit for study by an investigator

[Criteria rationale]

1)～4) Due to their impact on effectiveness evaluation

5)~11) For safety considerations

## Study method

This study will assess patients on warfarin who are scheduled for colorectal polypectomy, the target disease of this study. Patients will be allocated into a standard treatment group (heparin bridge group) or a trial treatment group (continued warfarin group) before starting treatment. Postoperative bleeding rates after endoscopic colorectal polypectomy will be compared. This is a prospective, multi-centre, two-group parallel, dynamically randomised, non-inferiority study.

## Target number of registered patients and study period

Case registration period: From the date of approval to August 31, 2022 (registration deadline August 31, 2022)

Total research period: From the date of approval to August 31, 2023

Registration period: 6 years.

Follow-up period: Up to 28 days after surgery.

Total research period: 7 years

List of definition of terms and abbreviations

Terms used in this study protocol

| Term | Definition |
| --- | --- |
| Postoperative haemorrhage | Observation of any of the following within 28 days after surgery will be defined as postoperative haemorrhage.  -Bloody stool with an Hb decrease of 2 g/dL or more.  -Overt bloody stool treated with endoscopic haemostasis, angiography, surgery, and/or blood transfusion. |
| Case requiring haemostasis during surgery | A case in which haemostasis techniques such as clipping was performed where spontaneous haemostasis did not occur. |
| Warfarin therapeutic range | PT-INR of 1.5 or more and 3.0 or less  Refer to the Gastrointestinal Endoscopy Practice Guidelines for Patients on Antithrombotic Drugs by the Japanese Gastroenterological Endoscopy Society, 2012 |
| ESD | Endoscopic submucosal dissection;  A method in which a lesion is collectively removed by injecting sodium hyaluronate solution locally into the submucosal layer of the tumour and incising and peeling using an electric scalpel |
| Hybrid ESD | A method in which the submucosal layer is separated to perform a snare at the end after making a perilesional incision using an ESD knife or a snare tip |
| FAS | Full analysis set: Defined as subjects who have been assigned to this study, have taken the study drug at least once, and have been evaluated for efficacy at least once after drug administration. |
| PPS | Per protocol set: Target population that meets the protocol requirements. |

List of abbreviations for general items

| Abbreviation | Unabbreviated expressions (Japanese) |
| --- | --- |
| NBI | narrow band imaging (狭帯域光観察) |
| AFI | autofluorescence imaging (自家蛍光観察) |
| SSA/P | sessile serrated adenoma/polyp (大腸鋸歯状腺腫/ポリープ) |
| LST | **laterally spreading tumours (**側方発育型腫瘍) |
| CRF | case report form (症例調査票) |
| POD | postoperative day (術後日数) |
| IRB | Institutional Review Board (治験審査委員会) |
| DOAC | direct oral anticoagulant drugs (直接経口抗凝固薬) |
| ASA-PS | [American Society of Anaesthesiologists](https://en.wikipedia.org/wiki/American_Society_of_Anesthesiologists) - physical status classification (米国麻酔学会術前状態分類) |

**Table of Contents**

[0 Overview i](#_Toc38214692)

[0.1 Schema i](#_Toc38214693)

[0.2 Purpose i](#_Toc38214694)

[0.3 Subjects i](#_Toc38214695)

[0.4 Study method iii](#_Toc38214696)

[0.5 Target number of registered patients and study period iii](#_Toc38214697)

[1 Study Background 1](#_Toc38214698)

[2 Study purpose 3](#_Toc38214699)

[3 System of Research Implementation 3](#_Toc38214700)

[4 Researcher names 3](#_Toc38214701)

[4.1 Research participating facilities (organisations), facility managers, facility coordinators * Alphabetical order (facility) 3](#_Toc38214702)

[4.2 Statistical analysis 5](#_Toc38214703)

[4.3 Institutional Review Board (IRB) of each facility 5](#_Toc38214704)

[5 Overview of Trial Drugs 6](#_Toc38214705)

[5.1 Trial Drugs 6](#_Toc38214706)

[5.2 Overview of equipment used 6](#_Toc38214707)

[5.3 Expected adverse reactions and medical device failures 6](#_Toc38214708)

[6 Target Disease and Macroscopic Classification 7](#_Toc38214709)

[6.1 Target disease 7](#_Toc38214710)

[6.2 Macroscopic Classification of Colorectal Polyp ^19)^ (Following the Japanese Classification of Colorectal, Appendiceal, and Anal Carcinoma, 8th edition, Japanese Society for Cancer of the Colon and Rectum) 7](#_Toc38214711)

[7 Study Subjects 8](#_Toc38214712)

[7.1 Inclusion Criteria 8](#_Toc38214713)

[7.2 Exclusion Criteria 8](#_Toc38214714)

[8 Study protocol 9](#_Toc38214715)

[8.1 Study Type/Design 9](#_Toc38214716)

[8.2 Case registration method 9](#_Toc38214717)

[8.3 Study outline 11](#_Toc38214718)

[8.4 Study participation period of subjects 15](#_Toc38214719)

[8.5 Usage and dose of trial drug, administration period 16](#_Toc38214720)

[8.6 Regulations regarding concomitant drugs (therapy) 16](#_Toc38214721)

[8.7 Method of dose reduction/discontinuation (trial drug adjustment method) (only when necessary) 18](#_Toc38214722)

[8.8 Management and distribution steps of the trial drug 18](#_Toc38214723)

[8.9 Information on medication instruction 18](#_Toc38214724)

[9 Observation items, test items, and schedule 18](#_Toc38214725)

[10 Discontinuation criteria for each subject 1](#_Toc38214726)

[11 Response in case of an adverse event 2](#_Toc38214727)

[11.1 Response to subjects when adverse events occur 2](#_Toc38214728)

[11.2 Report of serious adverse events 2](#_Toc38214729)

[12 Termination, cancellation and suspension of study 2](#_Toc38214730)

[12.1 Study termination 2](#_Toc38214731)

[12.2 Study cancellation and suspension 2](#_Toc38214732)

[13 Assessment parameters 3](#_Toc38214733)

[13.1 Primary assessment parameters 3](#_Toc38214734)

[13.2 Secondary assessment parameters: 4](#_Toc38214735)

[14 Aggregation of data 5](#_Toc38214736)

[15 Statistical analysis 5](#_Toc38214737)

[15.1 Data handling 5](#_Toc38214738)

[15.2 Effectiveness analysis 5](#_Toc38214739)

[15.3 Safety analysis 6](#_Toc38214740)

[15.4 Interim Analyses 6](#_Toc38214741)

[16 Target number of cases and rationale 6](#_Toc38214742)

[16.1 Target number of cases 6](#_Toc38214743)

[16.2 Rationale 6](#_Toc38214744)

[17 Study period 6](#_Toc38214745)

[18 Ethical matters 7](#_Toc38214746)

[18.1 Response to Guidelines and Declaration of Helsinki 7](#_Toc38214747)

[18.2 Ethics Committee approval 7](#_Toc38214748)

[18.3 Consent/Explanatory documents and information provision to participants 7](#_Toc38214749)

[18.4 Consideration for human rights (protection of personal information) 8](#_Toc38214750)

[18.5 Consideration for safety and disadvantages 8](#_Toc38214751)

[18.6 Response to inquiries 8](#_Toc38214752)

[19 Research expenses 9](#_Toc38214753)

[19.1 Study funds and conflicts of interest 9](#_Toc38214754)

[19.2 Costs borne by patients 9](#_Toc38214755)

[19.3 Compensation for health damage and insurance participation 9](#_Toc38214756)

[20 Saving records 10](#_Toc38214757)

[21 Release of study results 10](#_Toc38214758)

[22 Study organisation 10](#_Toc38214759)

[22.1 Principal researcher 10](#_Toc38214760)

[22.2 Participating facilities・Principal Investigators 10](#_Toc38214761)

[22.3 Clinical trial office 10](#_Toc38214762)

[22.4 Data centre 10](#_Toc38214763)

[22.5 Effectiveness and Safety Monitoring Committee 11](#_Toc38214764)

[23 Deviations or changes from the study protocol 11](#_Toc38214765)

[24 Monitoring/Quality Control of Data 11](#_Toc38214766)

[25 Audit 12](#_Toc38214767)

[26 References 12](#_Toc38214768)

[27 Attached documents／Attached figures and tables（as needed） 13](#_Toc38214769)

# Study Background

　In Japan, the age-adjusted prevalence of colorectal cancer (per 100,000 population) is on the rise with approximately 64.1 for males and 36.1 for females. According to the 2014 Ministry of Health, Labour and Welfare's vital statistics^1)^, the number of colorectal cancer deaths is close to 50,000 a year, and active measures are needed. An endoscopic removal of colorectal adenoma, a precursor of colorectal cancer, is considered an effective treatment to reduce colorectal cancer deaths^2) 3)^.

　Warfarin, a type of anticoagulant, is widely used for the treatment and prevention of thromboembolism (venous thrombosis, myocardial infarction, pulmonary embolism, cerebral embolism, slowly progressing cerebral thrombosis, among others). Endoscopic resection of colorectal polyp results in postoperative bleeding in 0.9-7% of cases, but the rate is reported to increase by about 10% in patients taking anticoagulants^4)^. On the other hand, discontinuing anticoagulants is considered to increase the risk of thrombosis to about 3%^5)^. Therefore, the guidelines for gastroenterological endoscopy in patients undergoing antithrombotic treatments, issued by the Japanese Society of Gastroenterological Endoscopy (JGES), recommend that patients discontinue anticoagulants and replace them with heparin, and perform endoscopic procedures. The American Society for Gastrointestinal Endoscopy (ASGE) guidelines also recommend heparin bridge in patients taking anticoagulants, a standard treatment at present. The JGES guidelines are based on the results of a case series of heparin bridged patients, previously on antiplatelets, whose upper and lower gastrointestinal endoscopy treatments did not develop any accidental contitions^6)^.

In contrast, in atrial fibrillation surgical cases where patients remained on warfarin without heparin bridge, the incidence of embolism was inferior to that of heparin bridge. Meanwhile, it was reported that with heparin replacement, the rate of haemorrhage increased^7)^. The incidence of haematoma in patients with pacemakers/implantable defibrillators was 16.0% in heparin bridge cases, significantly higher than those who remained on warfarin with a rate of 3.5%^8)^. In a meta-analysis study, patients who continued on warfarin was shown to have a significantly lower risk of postsurgical haemorrhage and haematoma than patients with heparin bridge, and the two groups did not show any difference in the embolism risk ^9)^. A meta-analysis of 207 studies on dental surgery, including tooth extraction, indicated that continued anticoagulants does not increase the risk of clinically significant bleeding compared to cases where anticoagulants were reduced or interrupted^10) 11)^.

The rate of bleeding after endoscopic polypectomy with heparin bridge is reported to be 20.0%^12)^. The postoperative bleeding rate was 12% in a retrospective study at the Department of Gastroenterology, Osaka City University School of Medicine. It has been reported that the frequency of postoperative bleeding is high in heparin bridged cases of colorectal polypectomy.

On the other hand, polypectomy in 70 patients who continued on warfarin did not show intraoperative or postoperative bleeding with haemostatic difficulties, and the postsurgical bleeding rate was 14% ^13)^. In addition, among patients with continuous warfarin, the rate of bleeding was 4.6% (4/87) in Cold polypectomy, whereby a lesion is removed without electric current, while the rate was 3.6% (2/56) with electric current. Difficulties with haemostasis and significant accidental conditions were not reported. ^14)^

The continued use of warfarin has also been reported in endoscopic submucosal dissection (ESD) of the stomach, with a higher rate of bleeding and perforation than colorectal polypectomy. The rate of postsurgical bleeding was 11.1% in the group that stayed on warfarin, and 21.7% in the heparin bridge group. The report suggested that the bleeding rate was lower in the group that continued on anticoagulants, without any significant accidental conditions^15)^.

　 In conducting this study, the risk of emergency surgery in the event of accidental conditions (perforation or bleeding where endoscopic haemostasis is impossible) may pose a problem in patients who continue warfarin. A case report of 13 renal transplant surgery cases with continued warfarin found that the rates of bleeding and embolism were similar to those in heparin bridged cases ^16)^. In addition, according to a nationwide questionnaire on accidental conditions from 2003 to 2007 by the Japanese Society of Gastroenterological Endoscopy, perforations associated with colorectal polypectomy were detected in 0.0235% (73/310584), and accidental conditions associated with endoscopic mucosal resection (EMR) were seen in 0.0636% (144/226562). Both combined, complications were relatively rare at 0.04% ^17)^, and the need of emergency surgery is extremely rare. The risk of emergency surgery may be higher than heparin bridged patients. However, we often experience emergency surgery in patients who have continued warfarin. Though lacking in evidence, some believe that bleeding control is possible by operating after PT-INR has been controlled by vitamin K supplementation and administration of fresh frozen plasma. It may therefore be possible to perform surgery while controlling for bleeding tendencies.

Thus, we hypothesised that colorectal polypectomy under continued anticoagulation may have a lower bleeding rate than heparin bridged cases, and that continuing warfarin is not inferior to heparin bridge. To date, no report has compared postoperative bleeding after endoscopic colorectal polypectomy in patients who stayed on anticoagulants to those who underwent heparin bridge^18)^. The problem with heparin bridge is that it requires a 24-hour continuous drip infusion. This means an extra burden on patients and medical staff, and that long-term hospitalisation is necessary due to additional time required by switching from warfarin to heparin before and after treatment. There is also a corresponding increase in medical costs. If the rate of bleeding after endoscopic polypectomy in patients with continued warfarin is found to be not inferior to that of heparin bridge, there is no need for long-term hospitalisation, and the associated cost and patient burden due to heparin bridge will decrease. A safer endoscopic colorectal polypectomy can be performed without concern for increased thrombosis risk associated with warfarin discontinuation.

　In this study, we compare the standard treatment of heparin bridge to the novel treatment of continuous warfarin. This study was designed with the aim to prove that endoscopic colorectal polypectomy under continuous warfarin is not inferior to the same procedure under heparin bridge, in terms of the postoperative bleeding rate.

Direct oral anticoagulant drugs (DOAC), a novel group of oral anticoagulants, will be excluded from this study since the rate of postoperative bleeding after endoscopic colorectal polypectomy has not been clarified.

# Study purpose

Compared with conventional method where patients on warfarin potassium (warfarin) switch to heparin sodium (heparin) for endoscopic colorectal polypectomy, this study was designed to demonstrate that the trial method of endoscopic colorectal polypectomy under continuous warfarin is not inferior to the conventional heparin bridge method in terms of the frequency of postoperative bleeding events.

# System of Research Implementation

This study is a multicentre, randomised controlled trial. The principal investigators and the clinical trial office are as follows. The assignment and data management will be outsourced to the data centre within the Osaka City University Hospital Center for Clinical Research and Innovation.

Exam Office

Department of Gastroenterology, Osaka City University Hospital

Tel：06-6645-2316、2317

e-mail：[m2043767@med.osaka-cu.ac.jp](mailto:m2043767@med.osaka-cu.ac.jp), yasuaki-75@med.osaka-cu.ac.jp

Names of Researchers

# Names of Researchers, etc.

## Research participating facilities (organizations), facility managers, facility coordinators

・Osaka City University Hospital Yasuaki Nagami, Masafumi Yamamura

・Yuri Kumiai General Hospital Hisatomo Ikehara

・Akita University Noboru Watanabe

・Asakayama General Hospital Yoshihisa Watanabe, Atsushi Noguchi

　　・Ikuwakai Memorial Hospita Kenji Adachi, Junichi Okamoto

・Ishikawa Prefectural Central Hospital

Hisashi Doyama, Shigetsugu Tsuji

・Ishikiriseiki Hospital Hiroaki Minamino, Yosuke Kinoshita

・Izumiotsu Municipal Hospital Takayuki Katsuno

　　・Osaka Medical Collage Toshihisa Takeuchi, Kazuki Kakimoto

　　・Osaka Ekisaikai Hospital Hironori Uno

　　・Osaka City General Hospital Hiroko Nebiki, Takehisa Suekane

・Okayama Medical Center Tomohiko Mannami, Toshiyuki Wakatsuki

・Okayama University Hospital Keita Harada, Yuusaku Sugihara

・Kanazawa University 　　　　Kazuya Kitamura, Hirotaka Yanase

・Kansai Rosai Hospital Shinjiro Yamaguchi, Takashi Ota

・Kindai University Faculty of Medicine

Hiroshi Kashida, Yoriaki Komeda

・Kyoto Second Red Cross Hospital

Takuji Kawamura

・Keio University School of Medicine

　　　　　　　　Motohiko Kato

・Kohnodai Hospital, National Center for Global Health and Medicine

Tomoyuki Yada

　　　　・Tonan Hospital　　　　　　　　Tetsuya Sumiyoshi

・Shiga University of Medical Science

　　　　　　　　Mitsushige Sugimoto

・Shizuoka Cancer Centre Kenichiro Imai

・Kashiwara Municipal Hospital Hiroyuki Sato, Masatsugu Okuyama

・Nara City Hospital 　　　　Takaaki Kishino, Kohei Fukumoto

・Chiba-Nishi General Hospital Satoshi Ono

・Takarazuka City Hospital Zhaoliang  Li

・University of Tsukuba  　　　　 Yuji Mizokami, Toshiaki Narasaka

・Tsuyama Chuo Hospital　　　　Ko Miura

・Tokyo Medical University Shin Kono

・Tokushima University Graduate School

　　　　　　　　Tetsuji Takayama, Shinji Kitamura

・Osaka International Cancer Institute

Yoji Takeuchi, Mitsuhiro Kono

　・Tottori University Hajime Isomoto, Koichiro Kawaguchi

・Toyama University Hospital Haruka Fujinami

・Dokkyo Medical University Kenichi Goda, Keiichiro Abe

　　　　・Nakae Hospital Mitsutaka Kumamoto

　　・Nagayoshi General Hospital Kenjiro Otani, Kazuki Yamamori

・Naniwa Ikuno Hospital Osamu Takaishi, Natsuhiko Kameda

・Nara Medical University Kei Moriya, Hideto Kawaratani

・Niigata University       　　　　 Shuji Terai, Satoru Hashimoto

・Osaka Red Cross Hospital Takeshi Yamashina, Manabu Fukuhara

・Japanese Red Cross Society Wakayama Medical Center

　　　 Takuji Akamatsu

・Nihon University Hospital Takuji Gotoda, Sho Suzuki

・Baba Memorial Hospital Junichi Hara, Taishi Sakai

・Hanwa Sumiyoshi General Hospital

Takashi Abe

・Higashisumiyoshi Morimoto Hospital

Koichiro Nakagawa, Masaki Takatsuka

・Hyogo College of Medicine Shiro Nakamura, Takako Miyazaki

・Hyogo Cancer Center 　　　　Yoshinobu Yamamoto

・Fukui Prefectural Hospital Yasuo Hashizume, Hiroyuki Aoyagi

・Mie University Hospital Yasuhiko Hamada

・Minami Osaka Hospital Takashi Fukuda, Masami Nakatani

・Meijibashi Hospital　　　　　　Masahiro Ochi

・Yamagata University Faculty of Medicine

　　　　　　　　Takao Yaoita, Yu Sasaki

・Shuto General Hospital Shu Kiyotoki

・Wakayama Medical University Mikitaka Iguchi

## Statistical analysis

Department of Medical Statistics, Osaka City University Graduate School of Medicine　　Hisako Yoshida

## Institutional Review Board (IRB) of each facility

In order to participate in this study, the study protocol and explanatory documents for patients must be approved by each institution's Ethics Committee or the Institutional Review Board (IRB). Any revisions to the study protocol and explanatory documents for patients during the study must be approved by each institution's Ethics Committee or the IRB. When approved by the IRB, the representative of each facility will send a copy of the approved document to the data centre. The data centre will accept case registrations from a facility once its IRB approval is confirmed.

# Overview of Trial Drugs

## Trial Drugs

Anticoagulants

Warfarin®: See package insert for details

Generic name: warfarin potassium, formulation: uncoated tablet, content: 0.5 mg tablet (warfarin potassium 0.5 mg/tablet), 1.0 mg tablet (warfarin potassium 1 mg/tablet), 5.0 mg tablet (warfarin potassium 5 mg/tablet), storage: store at room temperature, sold by: Eisai Co., Ltd., approved indications: treatment and prevention of thromboembolism (venous thrombosis, myocardial infarction, pulmonary embolism, cerebral embolism, slowly progressing cerebral thrombosis).

Heparin Na Injection®: See package insert for details

Generic name: Heparin sodium, formulation: colourless to pale yellow clear aqueous injection, content: 5000 units (heparin sodium 5000 units/vial), 10,000 units (heparin sodium 10,000 units/vial), storage: avoid sunlight, sold by: Mochida Pharmaceutical Co., Ltd., approved indications: treatment of generalised intravascular coagulation syndrome; prevention of blood coagulation during haemodialysis, cardiopulmonary bypass and use of other extracorporeal circulation devices; prevention of blood coagulation with vascular catheter insertion; blood transfusion and blood test; treatment and prevention of thromboembolism (venous thrombosis, myocardial infarction, pulmonary embolism, cerebral embolism, thromboembolism of the extremities, and intra- and postoperative thromboembolism)

Note: Warfarin/heparin preparations can be replaced by those used at each facility.

## Overview of equipment used

The electronic endoscope system and lower gastrointestinal endoscope regularly used in each facility will be used. The type of snare permitted is not specified, but it is to be noted in the CRF. Endoscopic colorectal polypectomy is performed according to the routine medical practice of each facility.

## Expected adverse reactions and medical device failures

Warfarin side effects include bleeding (intra-organ bleeding such as cerebral haemorrhage, mucosal bleeding, and subcutaneous bleeding), skin necrosis, and liver dysfunction. Heparin side effects include shock, anaphylaxis, bleeding, thrombocytopenia, heparin-induced thrombocytopenia/thrombosis, pruritus, hives, chills, fever, rhinitis, bronchial asthma, irritability, tearing, alopecia, vitiligo, haemorrhagic necrosis, and AST (GOT)/ALT (GPT) elevation.

In addition, this study uses the same medical devices as those used in regular clinical practice. Their safety and effectiveness have been established to date. There may be defects in endoscopy tools.

# Target Disease and Macroscopic Classification

## Target disease

Colorectal polyps preoperatively diagnosed to be resectable en bloc (adenoma, cancer, suspected SSA /P).

Even when there are concurrent lesions that are determined as unresectable en bloc or indicated for surgery, a polyp diagnosed as resectable en bloc is considered a target disease. Polyps for resection may be of any number, size, and macroscopic type.

　Note:・SSA/P (sessile serrated adenoma/polyp) is typically a pale, flattened and broad-based polypoid lesion with a slightly undefined border, often found in the right-sided colon.

## Macroscopic Classification of Colorectal Polyp ^19)^ (Following the Japanese Classification of Colorectal, Appendiceal, and Anal Carcinoma, 8th Edition, Japanese Society for Cancer of the Colon and Rectum)

**Subclassification of type 0 (superficial type)**

**I: Elevated**

**Ip: Pedunculated**

**Isp: Subpedunculated**

**Is: Sessile**

- II: Superficial

**IIa: Superficial elevated**

**IIb: Superficial flat**

**IIc: Superficial depressed**

**Note 1: In interpreting findings of a superficial macroscopic type polyp, endoscopic findings are prioritised. The entirety of the lesion is considered, regardless of histogenesis and difference between tumour and non-tumour tissue**.

**Note 2: Because it is difficult to distinguish adenoma from cancer by macroscopic findings, superficial type subclassification is also used for macroscopic classification of adenomatous lesions.**

**Note 3: For tumours with characteristics that belong to two categories, the category with a larger area is listed first, followed by a “+” and the other category.**

**Example: IIc+IIa**

**Note 4:** 0-IIa refers to a lesion with a height that does not exceed closed biopsy forceps (about 2.5 mm)**,** and any lesion with a greater height is classified as 0-Is**.**

# Study Subjects

Patients who underwent consultation at institutions participating in this study, who are taking warfarin in an outpatient or inpatient setting, and who are scheduled to undergo colorectal polypectomy.

## Inclusion Criteria

Patients who satisfy the following criteria are selected.

1. Patients with polyps that can undergo en-bloc resection of the large intestine (cecum, colon, and rectum), and who are scheduled for endoscopic colorectal polypectomy
2. Patients who have been taking warfarin for at least 2 weeks prior to endoscopic colorectal polypectomy
3. Patient is at least 20 years old at the time of obtaining consent
4. Written consent is provided based on the patient’s free will, after he or she has a thorough understanding of the explanation given regarding study participation

[Criteria rationale]

1) 2) Target patients who undergo endoscopic treatment while continuing to take anticoagulants.

3) 4) For safety considerations

Note 1) Patients on DOAC are excluded from this study since the rate of postoperative bleeding after endoscopic colorectal polypectomy has not been clarified.

Note 2) Included also are cases that are diagnosed as resectable en-bloc, but actually undergo piecemeal resection.

## Exclusion Criteria

Patients who meet any of the following criteria are excluded.

1. Patients with a history of enrolment in this study
2. Patients with inflammatory bowel disease, familial adenomatous polyposis, and Peutz-Jeghers syndrome
3. Cases whose clinical course cannot be followed up to 28 days after treatment
4. Any case with a history of bleeding with blood transfusion of 2 RBC units or more, Hb reduction of ≧ 2g/dL or haemostasis treatment within 6 weeks before surgery
5. Dialysis patients
6. Patients whose blood test show a platelet count of less than 50,000/μL within 12 weeks before surgery
7. Patients with coagulation dysfunctions
8. Pregnant patients
9. Lactating patients
10. Patient is allergic to heparin and warfarin
11. Other cases determined to be unfit for study by a head doctor or doctor in charge

[Criteria rationale]

1)~4) Due to their impact on effectiveness evaluation

5)~11) For safety considerations

# Study protocol

## 8.1 Study Type/Design

Prospective, multicentre, two-group parallel noninferiority study with dynamic randomisation

## Case registration method

8-2-1 Registration procedures

In this study, cases will undergo dynamic randomisation using the online registration allocation system at the data centre within the Osaka City University Hospital Center for Clinical Research and Innovation. The following three allocation adjustment factors will be used: (1) facilities, (2) the number of known lesions, and (3) concomitant use of antiplatelet drugs. For patients who provide written consent after receiving study explanations, we will confirm that they satisfy the inclusion criteria and that they do not meet any of the exclusion criteria.

The Principal Investigator or Co-Investigator will perform the following.

1) Before registration, online registration allocation system (Attachment 3) including the facility name, department name, contact information, and user name (Investigator at each facility) will be submitted via fax to the data centre.

2) The data centre will issue a user ID and password for each facility, required for online registration, by email.

3) Obtain written consent from the patient.

4) Access the online registration/allocation system via the Osaka City University Hospital Center for Clinical Research and Innovation website, and register the patient. Enter patient information (doctor in charge, patient identification code [ID], date of birth, date of consent), and confirm that the patient satisfies the inclusion criteria and that they do not meet any of the exclusion criteria. In facilities where reporting personal information (ID, date of birth, etc.) is restricted, the facility's patient identification code and the age of the patient at the time of obtaining consent will be recorded. The information will be stored by patient identification code at each facility.

5) Once registration is complete, the registration number and assigned group will be provided online.

- - No registration is allowed after the start of lower gastrointestinal endoscopy for the purpose of endoscopic colorectal polypectomy.
  - Once registered, no case registration will be removed. In case of duplicate registration, the initial registration information (registration number, allocation group) will be used in all cases.
  - If incorrect registration or duplicate registration is found, contact the data centre immediately.

8-2-2 Patient Registration

Inquiry

Osaka City University Hospital Center for Clinical Research and Innovation

TEL：06-6645-3443

Gastroenterology, Osaka City University Hospital

TEL：06-6645-2316、2317

e-mail：[m2043767@med.osaka-cu.ac.jp](mailto:m2043767@med.osaka-cu.ac.jp), [yasuaki-75@med.osaka-cu.ac.jp](mailto:yasuaki-75@med.osaka-cu.ac.jp)

Taishi Sakai, Yasuaki Nagami

8-2-3. Allocation method

The subjects will be assigned to each treatment group by the central registration method. This study will use the dynamic randomised allocation by minimisation in order to control for the number of colorectal polyps between the two groups and the background risk factors of bleeding. The following three allocation adjustment factors will be used in the minimisation technique: 1) facilities, 2) the number of polyps known in advance, and 3) concomitant use of antiplatelet drugs (or lack thereof). Researchers at participating facilities will not be informed of the detailed procedure of the randomised allocation method.

8-2-4. Blinding

This study will not be blinded.

## Study outline

Conducting Endoscopic polypectomy

・Obtain consent and register/allocate patients

- **Standard treatment group (heparin bridge group)**

　　At least 1-5 days prior to endoscopic colorectal polypectomy

　　・Patient is hospitalised at least 4 days before the procedure. Discontinue warfarin from 4 days prior to the procedure date, and start heparin bridge. For heparin bridge, continuous intravenous infusion of unfractionated heparin of 10,000 to 20,000 units will be given per day at a dose of approximately 200 U/kg/day. The dose will be controlled to keep APTT within 1.5-2.5 times the control. If the target range of APTT is not achieved after 20,000 units or more, consult the prescribing doctor, cardiologist, or cardiovascular surgeon to determine the dose. Even if the APTT on the day of the procedure does not fall within the target range as a result, the case is not excluded and the procedure will be completed as scheduled. The final heparin dose (number of heparin units per 24 hours) will be noted on the CRF.

　　　Note) Consent/registration may be obtained in the outpatient clinic prior to admission.

　On the day of the endoscopic colorectal polypectomy

　　・Measure APTT/PT in the morning of the treatment day and confirm that PT-INR is less than 1.5. Treatment day will be postponed if the PT-IRN is 1.5 or more on the scheduled date. If treatment is done once PT-INR of less than 1.5 is confirmed, it will not be considered a protocol cancellation. However, the protocol will be discontinued if treatment was done due to the circumstances of each facility. At this time, vitamin K preparations may induce thromboembolism and is not recommended. Any cancellation/postponement and the postponed date of treatment will be noted on the CRF.

　　・The half-life of intravenous heparin is 40–90 minutes. Since the onset and disappearance of heparin effects occur in a short period, it should be discontinued 3 hours before treatment. Heparin is restarted as soon as the patient returns to the ward from the endoscopic treatment.

　　　*Note) See below for steps of pre-treatment to polypectomy

From the following day after endoscopic colorectal polypectomy

　　・Resume meals if there is no sign of bleeding such as bloody stools, decrease in Hb, and fluctuating vital signs.

・Do a blood test on the following day and measure blood count (white blood cells, red blood cells, haemoglobin, haematocrit, platelets), biochemistry (BUN, Cre, AST, ALT, T-bil, Na, K, Cl, CRP), PT-INR, and APTT.

・If there is no sign of bleeding as indicated above by the next morning, resume warfarin. Resume the same amount of warfarin that the patient received before discontinuation. After that, confirm that PT-INR has reached the therapeutic range (1.5 or more) by a blood test at least once every 3 days, and discontinue heparin. Patients can be discharged after heparin is discontinued. If reaching therapeutic range PT-INR takes a long time, patients may be discharged after discontinuing heparin when such approval is given by the prescribing physician, cardiologist, or cardiovascular surgeon. The final measurement of PT-INR will be noted in the CRF.

**Note: Warfarin/heparin preparations can be replaced by those used at each facility.

- **Trial treatment group (continued warfarin group)**

At least 1–5 days prior to endoscopic colorectal polypectomy

　　・Patients with PT-INR >3 on the day of admission will be treated once PT-INR has improved to 3 or less by adjusting warfarin.

　　・Patients will continue on outpatient warfarin dose. If assigned to the trial treatment group, the treatment date can be changed to an earlier date.

　　　Note) Consent/registration may be obtained in the outpatient clinic prior to admission.

The day of endoscopic colorectal polypectomy

　　・Measure APTT/PT in the morning of the treatment day and confirm that PT-INR is 3 or below. The treatment day will be postponed if PT-INR is >3 on the scheduled date. If treatment is done once PT-INR of 3 or below is confirmed, and it will not be considered a protocol cancellation. However, the protocol will be discontinued if treatment was done due to the circumstances of each facility. At this time, vitamin K preparations may induce thromboembolism and are not recommended. Any cancellation/postponement and the postponed date of treatment will be noted on the CRF.

　　・Continue warfarin on the day of treatment as well with the outpatient dose.

　　　*Note) See below for steps of pre-treatment to polypectomy

From the following day after endoscopic colorectal polypectomy

　　・Resume meals if there is no sign of bleeding such as bloody stools, decrease in Hb, and fluctuating vital signs.

・Do a blood test on the following day and measure blood count (white blood cells, red blood cells, haemoglobin, haematocrit, platelets), biochemistry (BUN, Cre, AST, ALT, T-bil, Na, K, Cl, CRP), and PT-INR.

・Continue warfarin from the following day. Patients may be discharged from the following day.

**Note: Warfarin/heparin preparations can be replaced by those used at each facility.

*** Note) Steps from pre-treatment to polypectomy (common to both groups)**

・Pre-treatment method

　　　Follow the pre-treatment at each facility's regular medical practice. Below, we list what may be regarded as a standard pre-treatment method. This is not intended to limit the method taken at each facility. We will take appropriate measures to allow this study to take place under the best possible conditions.

The day before the test: No dietary restrictions. Take 2 tablets of oral sennoside laxative (Plzenide) or a similar laxative. In a case of constipation, other drugs may be added.

　　　Test day: Take sodium/potassium combination agent powder (Nifrec), magnesium citrate (Magcorol P) or sodium/potassium/ascorbic acid combination agent powder (Mobiprep) (additional treatment may apply depending on the state of washing) as appropriate.

　　　Immediately before or during the test: Butylscopolamine (Buscopan) or glucagon may be used as antispasmodics.

　　　When using sedation, we will refer to the drugs recommended in the “combination therapy/supportive therapy” from the guidelines supervised by the Japanese Society of Gastroenterological Endoscopy. During sedation, a patient's condition will be monitored using the oxygen saturation monitor, among others.

　　・Endoscopy exam

　　　At the time of observation: Carefully collect the intestinal tract residue and intestinal fluids before observation. Carefully separate and observe each fold so that there are no oversights. The combined use of lesion detection tools such as NBI, AFI, dye observation, and transparent tip hood will not be specified.

　　・For endoscopic colorectal polypectomy in this study, the procedure will be performed by, or under the guidance of, a specialist physician qualified by the Japan Gastroenterological Endoscopy Society.

　　・Steps of endoscopic colorectal polypectomy

　　　The procedure will follow the following protocol

　　・Submucosal injection can be performed, and the type of injection will not be specified. It will be completed following regular clinical practice.

・The electric current setting and the type of snare used will not be specified. Each facility will follow its regular clinical practice and note the type of snare used on the CRF. Both bipolar and monopolar snares may be used.

・Do not perform cold polypectomy.

・Not all polyps need to be removed.

・After polypectomy, the resection site should be thoroughly checked. If an active bleeding without spontaneous haemostasis or exposed blood vessels is observed, additional haemostasis techniques such as clipping should be performed.

・After polypectomy, the resection site will be prophylactically clipped as a general rule in all cases. If clipping is not performed due to piecemeal excision, state the reason on the CRF.

・Nothing per os on the day of the treatment. Any fluid replacement will be determined by the head doctor.

In case of postoperative bleeding

　　・Obtain blood count, biochemistry, and PT-INR/APTT (APTT does not have to be measured in the warfarin group).

・Emergency lower gastrointestinal endoscopy will be performed to stop the source of bleeding in the following cases: twice or more samples of persistent bloody stool, without sign of improvement; bloody stool with changes in vital signs (systolic blood pressure <100 mmHg or pulse >90 beats/min); or bloody stool with an Hb decrease of 2 g/dL or more. Even if there is no active bleeding and the source of bleeding is unknown during urgent endoscopy, haemostasis techniques (additional clipping) will be performed if haemorrhage is strongly suspected (adhesion of blood clots or exposed blood vessels). For the bleeding source or an area with high suspicion for haemorrhage, the lesion number, location, the presence or absence of haemostasis, and the haemostasis technique will be noted in the CRF. If endoscopic haemostasis is difficult, the following will be noted in the CRF: the presence or absence of angiography, the presence or absence of surgery, the presence or absence of blood transfusion, and the number of units. If haemostasis can be confirmed, the patient may or may not require hospitalisation.

　　・If PT-INR >3, patient management will be consulted with the prescribing physician, cardiologist, or cardiovascular surgeon. Warfarin may be discontinued at the time.

　　・If a patient is still on heparin, continue heparin administration/oral administration of warfarin.

In case of rebleeding, warfarin may be discontinued after consultation with the prescribing physician, cardiologist, or cardiovascular surgeon. If bleeding persists, consider stopping heparin as well. If heparin administration is discontinued, the trial treatment group protocol will apply.

・In case of repeated rebleeding, heparin/warfarin may be discontinued at the discretion of the head doctor after consultation with the prescribing physician, cardiologist, or cardiovascular surgeon. The discontinuation date should be indicated on the CRF.

・If the patient was also taking an antiplatelet drug, whether or not the drug was withdrawn will also be indicated on the CRF.

Cases scheduled for endoscopic colorectal polypectomy

Restart heparin

Endoscopic colorectal polypectomy

Discontinue heparin

Warfarin continued

Standard treatment group

Discontinue heparin

Heparin + Warfarin

Obtain consent/randomised allocation

Warfarin discontinued

heparin bridge

Trial treatment group

Warfarin continued

The day after treatment

After return to the ward

3 hours before treatment

4 days before planned treatment day

Not specified

## Study participation period of subjects

　　　Until follow-up on postoperative day 28 for all cases

　　　Patients should ideally be questioned for the presence of any adverse events at an outpatient clinic 28 days post-surgery. If this is difficult, patients should be contacted by telephone to check for any adverse events, which must be reported and noted on the CRF should they occur. In addition, patients should be advised to seek medical attention in case of a bloody stool. Blood count and PT-INR are measured on postoperative day 28. If a blood test cannot be done on postoperative day 28, it can be done up to postoperative day 35. If a patient is unable to come to the outpatient clinic on postoperative day 28, the patient may visit between postoperative day 14 and 27. In such cases, a phone call will also be made after postoperative day 28 to check for any adverse events. If an adverse event occurs between the outpatient clinic visit and postoperative day 28, it should always be reported and noted on the CRF.

## Usage and dose of trial drug, administration period

　　Follows 8.3.

## Regulations regarding concomitant drugs (therapy)

1. Concomitant medication (therapy):

・Antiplatelet drugs

If the subject was on an antiplatelet drug prior to administration of the trial drug, the Principal Investigator will note the name of the drug, whether or not the drug was withdrawn, the duration of drug withdrawal, the reason for use (thromboembolism high-incidence group/low-incidence group), and the presence or absence of substitution in the CRF.

Aspirin monotherapy: Aspirin will be continued in patients in the high thromboembolism incidence group. That is, there will be no drug interruption including the day of surgery and postoperative day 1. In the low thromboembolism incidence group, withdrawal/continuation of a drug is possible at the discretion of the doctor in charge. If the drug is withdrawn, it will be restarted the day after surgery.

Ticlopidine (Panaldine)/clopidogrel (Plavix)/prasugrel (Effient): Withdraw drug from 5 days prior, and resume oral administration from the day after surgery. Substitute with aspirin or cilostazol (pletal) in patients with high incidence of thromboembolism.

Antiplatelet drugs other than ticlopidine/clopidogrel: Discontinue from the day before surgery. Resume oral administration the day after surgery.

Aspirin and other antiplatelet agents: Aspirin will be given continuously. Ticlopidine/clopidogrel will be discontinued from 5 days before, and oral administration will be resumed from the day after surgery. Antiplatelet drugs other than ticlopidine/clopidogrel will be discontinued from the day before surgery, and oral administration will be resumed the day after surgery.

- The high thromboembolism incidence group corresponds to patients with the following.
- Within 2 months of coronary stent placement.
- Within 12 months of drug-eluting stent placement in coronary arteries.
- Within 2 months of cerebral revascularisation (carotid endarterectomy and stent placement).
- Cerebral infarction or transient ischaemic attack with 50% or greater stenosis in the main arteries.
- Recent ischaemic stroke or transient ischaemic attack.
- Arteriosclerosis obliterans with a Fontaine Stage III (pain at rest) or higher.
- Findings in carotid artery ultrasonography or head and neck magnetic resonance angiography that indicate a high risk of drug discontinuation

・Combination drugs during endoscopic colorectal polypectomy

The following combination and supportive therapies are accepted. Not following these therapies will not be considered a protocol deviation.

Antispasmodics: Butylscopolamine (Buscopan), glucagon, etc.

Sedatives and analgesics: Flunitrazepam (Silase, Rohypnol), diazepam (Cercine, Horizone), midazolam (Dolmicum), propofol (Diprivan), pethidine hydrochloride (Opistan), pentazocine (Sosegon, Pentadine), etc.

・Reefing method with clipping for perforation during endoscopic colorectal polypectomy

If there is perforation during endoscopic colorectal polypectomy, endoscopic reefing with clipping will be immediately performed.

・Prophylactic administration of haemostatic agent after endoscopic colorectal polypectomy

Use of thrombin (topical application) and sodium carbazochrome sulfonate (Adna) will be accepted.

1. Prohibited combination drugs (therapy):

・Tranexamic acid (Transamine)

・While the patient is being treated according to the protocol, any treatment outside the protocol such as surgery, chemotherapy, radiation therapy, and immunotherapy will not be performed.

## Method of dose reduction/discontinuation (trial drug adjustment method) (only when necessary)

During hospitalisation:

・If PT-INR >3, consider reducing warfarin on consultation with a prescribing physician, cardiologist, or cardiovascular surgeon.

Standard treatment group:

・If APTT increases to 2.5 times or more than the level prior to administration, the dose will be reduced by 2,500-5,000 units. If the APTT does not reach 1.5 times the level before administration, the dose will be increased by 2500–5000 units. When exceeding 20,000 units, the dose will be decided on consultation with the prescribing physician, cardiologist, or cardiovascular surgeon.

-If PT-INR >3 after restarting warfarin, consider reducing warfarin in consultation with a prescribing physician, cardiologist, or cardiovascular surgeon.

-If PT-INR does not reach the therapeutic range after restarting warfarin, consider increasing the dose in consultation with the prescribing doctor, cardiologist, or cardiovascular surgeon.

Trial treatment group:

・Continue warfarin the day after surgery and discharge patient on or after postoperative day 1.

In either group, if an Investigator determines that the withdrawal of the trial drug is necessary due to postoperative bleeding after endoscopic colorectal polypectomy; the trial drug should be discontinued and stated as such on the CRF.

## Management and distribution steps of the trial drug

The drug will be supplied by the pharmacy with a prescription.

## Information on medication instruction

Nothing in particular.

# Observation items, test items, and schedule

The following information will be collected according to the schedule.

1. Patient background

Medical record number (ID number), sex, date of birth (age), surgical history, oral dose of warfarin, disease names for which anticoagulants are prescribed (atrial fibrillation, venous thrombosis, myocardial infarction, pulmonary embolism, cerebral embolism, coronary artery bypass surgery, artificial valve replacement surgery, and others)

Comorbidity (diabetes, hypertension, heart disease, respiratory disease, renal disease, liver disease, brain disease, endocrine/metabolic disease, autoimmune disease, malignancy, and others)

ASA-PS

Note 1) In facilities where reporting personal information (ID and date of birth) is restricted, the facility's patient identification code and the age of the patient at the time of consent will be recorded. This information will be stored by patient identification code at each facility.

Note 2) ASA-PS ([American Society of Anesthesiologists](https://en.wikipedia.org/wiki/American_Society_of_Anesthesiologists) - Physical status classification)

　　Class 1: Healthy patients (except conditions requiring surgery) (inguinal hernia, uterine fibroids, appendicitis surgery, among others)

　　Class 2: Patients with mild to moderate systemic disease

　　　Mild diabetes, mild essential hypertension, anaemia, neonates and those who are 80 years or older, severe obesity, chronic bronchitis, other tumours, etc.

Class 3: Patients with severe systemic disease

Severe diabetes mellitus, moderate/advanced lung disease (COPD), controlled ischaemic heart disease (history of PCI/CABG), cerebral infarction, liver cirrhosis (Child-Pugh class B/C), dialysis, multiple malignancy metastases, etc.

Class 4: Patients with severe life-threatening systemic diseases

Multiple organ failure, and others.
Class 5: Near-death patients who cannot survive without surgery

Shock due to myocardial infarction, ruptured aortic aneurysm, severe pulmonary embolism, etc.
Class 6: Brain dead patients

1. The presence or absence of antiplatelet drug administration

If a patient was on an antiplatelet drug prior to trial drug administration, the Principal Investigator will note the drug name, the presence or absence of drug withdrawal and its duration, reason for antiplatelet drug use (thromboembolism high risk group/low risk group), and whether or not the drug was replaced on the CRF.

1. Check for subjective symptoms and objective findings

Confirm by patient interview. If available, a symptom diary may be referred.

1. Observation of lesions

Deepest insertion point, the presence or absence of colorectal surgery, number of lesions, lesion number, lesion size, macroscopic type, tissue type, lesion site, the presence or absence of immediate postsurgical bleeding, treatment method (polypectomy/EMR), the type of local injection solution, number of clippings performed after resection, whether or not the lesion was collected, snare type (bipolar/monopolar), the presence or absence of immediate postsurgical haemorrhage without spontaneous haemostasis, the presence or absence of perforation during treatment, treating physician's years of experience

Note) Histological type will be confirmed during the outpatient visit after discharge

1. Check for adverse events and side effects
2. The presence or absence of bloody stool
3. Haematology test

Peripheral blood count: white blood cells, red blood cells, haemoglobin, haematocrit, and platelets

1. Blood biochemistry test

Renal function: BUN, Cre

Liver function: AST, ALT, T-bil

Electrolytes: Na, K, Cl,

Inflammatory reaction: CRP

1. PT-INR
2. APTT

In case of postsurgical bleeding, re-examine (7), (8), (9), and (10). The measurement of APTT with postoperative bleeding is not required in the trial treatment group.

Schedule

| Period | Outpatient | 5~1 day prior to admission date | 4 to 1 day before planned treatment date | Treatment date | The day after treatment |  | Postoperative bleeding | Postoperative day 28 |
| --- | --- | --- | --- | --- | --- | --- | --- | --- |
| Patient visit | Visit１ | Admission | Admission | Admission | Admission | Admission →  Discharge | Emergency visit | Visit 2 |
| Obtain consent | ○ | |  |  |  |  |  |  |
| Patient background check | ○ | ● |  |  |  |  |  |  |
| Subjective symptoms/Objective findings | ○ | ● |  | ○ | ○ | ○ | ○ |  |
| Examination  (Physical findings/ Observe adverse events) | ○ | ● |  | ○ | ○ | ○ | ○ |  |
| Antiplatelet drug administration check | ○ | ● |  |  |  |  |  |  |
| Adverse event and side effect check |  |  | ● | ● | ● |  |  |  |
| Presence or absence of bloody stool |  |  |  |  | ○ | ○ | ○ |  |
| Warfarin | ○ | ○ | Discontinue in heparin bridge group Continue in warfarin continued group | | ○ | ○ | ○ |  |
| Heparin bridge |  |  |  |  |  |  |  |  |
| Endoscopy exam |  |  |  | ○ |  |  | ○ |  |
| Lesion observation |  |  |  | ○ |  |  |  |  |
| Haematology test | ○ |  |  |  | ○ |  | ○ | ○ |
| Biochemistry test | ○ |  |  |  | ○ |  | ○ |  |
| PT－INR | ○ | ○ | ● | ○ | ○ | ● | ○ | ○ |
| APTT | ○ | ● | ● | ● | ● | ● | ● |  |

○ indicates items that must be implemented; ● indicates items that will be implemented as necessary

Note) Consent can be obtained in the outpatient setting as well. In addition, a patient can be treated if the PT-INR becomes 3 or less after hospitalisation.

Note) If a blood test cannot be done on postoperative day 28, it can be done up to postoperative day 35.

# Discontinuation criteria for each subject

1. Response in case of research discontinuation

If a researcher determines that a subject cannot continue to participate in research for the following reasons, the participation of that subject will be cancelled. In such cases, the subject will receive an explanation for discontinuation, as necessary. The treatment of a subject after study cancellation should be done with integrity so as not to put him/her at any disadvantage.

(2) Cancellation criteria

If any of the following criteria are met, the protocol treatment will be discontinued. The reason for discontinuation will be stated in the medical chart and CRF, and the CRF will be promptly reported by mail to the data centre within the Osaka City University Hospital Center for Clinical Research and Innovation.

1. **Standard treatment group (heparin bridge group);** Cases with PT-INR >1.5 (on study day): However, if the treatment is postponed and the treatment is performed once the PT-INR is 1.5 or less, it will not be considered a protocol cancellation.
2. **Trial treatment group (continued warfarin group);** Cases with PT-INR >3 (on study day): However, if the treatment is postponed and the treatment is performed once the PT-INR is 3 or less, it will not be considered a protocol cancellation.
3. Cases of ESD and Hybrid ESD
4. Cases where endoscopic colorectal polypectomy is cancelled before completion of the procedure (the procedure will be deemed complete with the constriction of a snare)
5. If a gastrointestinal perforation is seen during or after endoscopic colorectal polypectomy
6. When an additional surgery is performed within 28 days after endoscopic colorectal polypectomy
7. When a subject requests to leave the study or withdraws consent
8. Other reasons given by a subject (too busy, transfer to another hospital, relocation, etc.)
9. If a subject is found to not satisfy the eligibility criteria after registration
10. When continuing the study is difficult due to worsening complications
11. When continuing the study is difficult due to adverse events
12. If a physician decides that cancelling the study is appropriate for any other reasons

Note) Hybrid ESD: A technique using an ESD-specific knife or a snare tip. After making an incision around the lesion, the submucosal layer will be separated to complete snaring

# Response in case of an adverse event

## Response to subjects when adverse events occur

・Adverse events include all unfavourable or unintentional injuries, illnesses, or symptoms (including abnormal laboratory test values) that occur in subjects, regardless of their causal relationship with the study conducted.

・If an adverse event is observed, a researcher will immediately take appropriate measures and record it in the medical records. Subjects will be informed when the administration of trial drug or the use of test equipment is discontinued, or if treatment for an adverse event is required.

## Report of serious adverse events

　A "serious adverse event" refers to any unfavourable medical event regardless of dose that meets the following conditions.

(1) Causes patient death

(2) Life threatening

(3) Requires hospitalised treatment or extended hospitalisation

(4) Causes permanent or marked disability/dysfunction

(5) Causes congenital malformation in an offspring

If a Principal Investigator encounters a serious adverse event during an invasive study, he/she should promptly report all such cases to the hospital director regardless of the causal relationship with the study. If a causal relationship between the event and the study cannot be ruled out in a multicentre trial, he/she must also report to the responsible physician of the relevant medical institution involved in the trial.

# Termination, cancellation and suspension of the study

## Study termination

　At the end of the study at each institution, the Principal Investigator will promptly submit a study completion report to the hospital director.

## Study cancellation and suspension

　The Principal Investigator will consider cancellation or suspension of the study if any of the following matters apply.

1) Important information regarding the quality, safety, and efficacy of the trial drug becomes available.

2) Study subject recruitment is difficult and reaching the planned number of cases is found to be nearly impossible.

3) When the study purpose is achieved (by interim analysis) before reaching the planned number of cases or study period.

4) When the Advisory Committee instructs to change the implementation plan that is determined difficult to accept.

The study will be cancelled if an Advisory Committee recommends or orders to do so. If several institutions are involved, the aforementioned matters will be examined by the principal researcher or the committee specified in the study protocol, and will determine whether or not to continue the study.

If it is decided to cancel or discontinue the study, this should be promptly reported in writing to the hospital director with explanations.

# Assessment parameters

## Primary assessment parameters

　Postoperative bleeding rate　Cases with postoperative bleeding/cases that underwent polypectomy

[Criteria rationale]

If the rate of postoperative bleeding with continued warfarin is not inferior to that of heparin bridge, endoscopic colorectal polypectomy with continuous warfarin could become the standard treatment. This leads to reduced burden associated with the heparin bridge on those involved in care and patients, and reduced cost.

Postoperative bleeding　When bleeding is observed with one or more of the following within 28 days after surgery

-Bloody stool with an Hb decrease of 2 g/dL or more.

- Overt bloody stool treated with endoscopic haemostasis, angiography, surgery, and/or blood transfusion.

Note) Even if there is no active bleeding and the source of bleeding is unknown during urgent endoscopy, haemostasis techniques (additional clipping) will be performed if haemorrhage is strongly suspected (adhesion of blood clots or exposed blood vessels).

Emergency endoscopy criteria

Emergency lower gastrointestinal endoscopy will be performed in the following cases: twice or more of persistent bloody stool without a sign of improvement; bloody stool with changes in vital signs (systolic blood pressure <100 mmHg or pulse >90 beats/min); or bloody stool with an Hb decrease of 2 g/dL or more.

Postoperative bleeding rate after endoscopic colorectal polypectomy will be compared between the two groups within the full analysis set (FAS) as defined in 15.2. As a complement, it will also be done within the per protocol set (PPS).

## Secondary assessment parameters:

1. Cumulative bleeding rate
2. Rate of overt haemorrhage that does not satisfy the definition of haemorrhage after endoscopic polypectomy
3. Incidence rate of haemorrhage that required haemostasis during endoscopic polypectomy

Cases that required haemostasis during surgery: Cases where a haemostasis technique such as clipping was performed for bleeding that did not undergo spontaneous haemostasis.

1. Intraoperative bleeding during endoscopic colorectal polypectomy requiring angiography, surgery and/or blood transfusion
2. Total bleeding rate (postoperative bleeding + ② + ③)
3. Risk factors for postoperative bleeding
4. Number of hospitalisation days
5. Incidence rate of thromboembolism
6. PT-INR 28 days after surgery (If it is difficult to perform a blood test on postoperative day 28, it can be performed up to postoperative day 35).
7. Percentage of serious adverse events

[Criteria rationale]

- - 1. To clarify whether there is a difference in the postprocedural bleeding rate, as well as timing of bleeding, between warfarin continued cases and heparin-bridged cases.
    2. For bleeding that did not require emergency treatment, we aim to clarify whether there is a difference in bleeding rate between warfarin continued cases and heparin bridged cases.
    3. With regard to intraoperative bleeding, we aim to clarify whether there is a difference in bleeding rate between warfarin continued cases and heparin-bridged cases.
    4. To clarify whether severe intraoperative bleeding that requires aforementioned treatment can occur in warfarin continued and heparin replaced cases, and if so, whether there is a difference in bleeding rate.
    5. All treatment-related bleeding will be assessed to clarify the difference between the warfarin continued group and the heparin-bridged group.
    6. This study is a non-inferiority study. However, we also aim to clarify whether warfarin administration or heparin bridge may be a risk factor for postoperative haemorrhage, and whether there are other risk factors for postoperative haemorrhage.
    7. Heparin-bridged patients are expected to have a longer hospital stay than those who continued to take warfarin.
    8. To clarify whether the incidence of thromboembolism in heparin-bridged patients differs from that in warfarin continued patients.
    9. To clarify whether PT-INR returns to preprocedural levels within 28 days in heparin bridged group, since it may affect the incidence of thromboembolism and postoperative bleeding.
    10. Because the lower the incidence of serious adverse events between the two groups, the better.

Regarding the above ② to ⑨, both groups will be compared with the full analysis set (FAS) as specified in 15.2, and per protocol set (PPS) will also be done complementarily.

# Aggregation of data

Data will be aggregated after collection of all cases.

# Statistical analysis

## Data handling

　Data obtained by a method that deviates from the study protocol will be assessed by medical experts. In addition, they will also discuss how to handle issues not specified during the planning stage.

## Non-inferiority analysis

Before describing the analysis method, we will define the target analysis group.

　The target analysis group will be set as the FAS, defined as subjects who were assigned to this study, who took the study drug at least once and were evaluated for efficacy at least once after study drug administration. Further analysis of PPS that meets the study protocol will also be done complementarily.

The primary assessment parameter of postoperative bleeding rate will be analysed using the Chi-squared test (Fisher's exact test) (one-sided test) and also by the 95% confidence interval.

For other analyses, describe in the statistical analysis plan separately defined.

## Safety analysis

A frequency table by category will be created regarding the occurrence of adverse events.

## Interim Analyses

To examine whether the study can be continued or not, an interim analysis will be conducted 28 days after the registration of 100 cases. The main purpose of the interim analyses will be to confirm the safety of the protocol’s treatment. In this case, only serious adverse events are evaluated and not primary assessment parameters.

# Target number of cases and rationale

## Target number of cases

Target number of cases: 316 cases; Osaka City University Hospital: 90 cases

## Rationale

　　　Few reports have examined the post-procedural bleeding rate after endoscopic polypectomy among patients who continued warfarin. According to a report by Horiuchi et al.^12)^, the rate of postoperative bleeding was 14% among those who continued warfarin, and 20% in patients who switched from warfarin to heparin. Based on these findings, the bleeding rate in this study can be assumed to be similar. We assume the post-procedural bleeding rate of 14% for warfarin continued cases, and 20% for heparin bridged cases. Non-inferiority margin was set to 5%. Given α value of 0.05 and power of 0.8, we considered an enrolment of about 144 cases in each group to be appropriate. The target number of cases was set at 158 in each group, for a total of 316, assuming that a little less than 10% of cases may be discontinued or are ineligible.

# Study period

Case registration period: From the date of approval to August 31, 2022 (registration deadline August 31, 2022)

Total research period: From the date of approval to August 31, 2023

Registration period: 6 years.

Follow-up period: Up to 28 days after surgery.

Total research period: 7 years

# Ethical matters

## Response to Guidelines and Declaration of Helsinki

This study will be conducted in strict compliance with the Ethical Guidelines for Medical and Health Research Involving Human Subjects (Ministry of Education, Culture, Sports, Science and Technology, established December 22, 2014) and the Declaration of Helsinki.

## Ethics Committee approval

This study is conducted with the approval of the Advisory Committee of Osaka City University Hospital.

## Consent/Explanatory documents and information provision to participants

Patients will receive the explanation and consent forms approved by the Ethics Committee. A complete written and verbal explanation will be given to the patient, and written consent will be provided based on the patient's free will.

Patients should be promptly advised in situations where their consent may be affected: such as when efficiency- and safety-related information become available, or when there is a change in implementation plan. Under such circumstances, the subject's intention regarding study participation should be confirmed in advance. These must be pre-approved by the Ethics Committee, and revised consent forms/explanatory documents must be obtained. With that, patient consent must be obtained once again.

The following items will be included in the explanatory form.

1. Introduction: Voluntary clinical trials

2. Your medical condition

3. Purpose of this clinical trial

4. Method of this study

5. Scheduled participation period of this study

6. Expected benefits and risks

7. Treatment details if you do not participate in this study

8. Treatment costs

9. Situations in which your study participation may be cancelled

10. Things you will need to follow upon agreeing to participate in this study

11. If your health is compromised during this study

12. Intellectual property rights and conflicts of interest of this research

13. Participation in this test is at your own discretion

14. Information about this study will be provided to you as they become available

15. Your privacy will be protected even when results are made public

16. Your medical records may be investigated during or after the study by participating in this study

17. Preservation of materials (including samples), use or disposal after research

18. Help desk

## Consideration for human rights (protection of personal information)

In implementing this study, efforts will be made to ensure that human rights, welfare, and safety of study participants are maximally protected. The Principal Investigator or Co-Investigator will carefully consider ethical issues when conducting this study, fully explain the content of the study, and obtain written consent from patients. Whether or not to participate in the study is based on the subject's free will. A patient can withdraw his or her consent at any time, even after giving consent. In addition, a patient will not be put at any disadvantage for withdrawing from the study. The information obtained from this study will not be published under any circumstances in an individually identifiable form, and will be stored under strict control. In addition, if a subject so desires, his or her information may be released in writing to the subject only.

## Consideration for safety and disadvantages

When an adverse event occurs, appropriate medical examination and treatment will be promptly performed. In case of a bleeding event, urgent endoscopic haemostasis, and blood transfusion will be performed as soon as possible. If bleeding persists without improvement, discontinuing warfarin/heparin will be considered in consultation with the prescribing physician, cardiologist, or cardiovascular surgeon. In addition, continued oral administration of warfarin can expect to reduce the risk of thrombosis. Compared to heparin-bridged cases, we can expect shortened hospital stay and subsequent reduction in costs.

## Response to inquiries

Consultations, inquiries, and complaints from study subjects or their legal representatives and other related persons will be handled with prompt and appropriate responses.

# Research expenses

## Study funds and conflicts of interest

This study is supported by a grant from the Japanese Gastroenterological Association. Eisai Co., Ltd. and Mochida Pharmaceutical Co., Ltd., that manufacture and sell the trial drug warfarin and heparin, respectively, do not provide any funds or benefits to this study. The planning and implementation of this study will be done by the researchers independently of these companies.

Research expenses will be managed by the financial accounting system of Osaka City University. For conflicts of interest, we will obtain approval from the Osaka City University Conflict of Interest Management Committee. By regularly updating our research (trial) progress to this committee, we will maintain fairness with regards to the conflicts of interest. We will ensure that the planning, implementation, and reporting of this study do not affect the outcome of the study or its interpretation. We will also ensure that the implementation of this study does not undermine the rights or interests of the study participants.

## Costs borne by patients

This study is conducted entirely within the scope of insurance-covered treatment. Drugs or tests that are not covered by insurance will not be administered. In addition, the number of hospital visits and the frequency of tests will be similar to those in regular medical treatment. We do not expect any additional burden on the patient due to study participation.

## Compensation for health damage and insurance participation

All drug administrations and tests in this study are done within the scope of insurance-covered treatment. Even if a health hazard occurs due to this study, the patient's health insurance will be used for treatment in the same manner as a regular insurance-covered treatment. Neither the organisation/facility involved in this research nor individuals such as a physician will cover medical co-payments, sick leave compensations, or extra bed charges, etc. However, the doctor in charge will take prompt and appropriate action to ensure that the best treatment is provided throughout the hospital. In carrying out this clinical trial, we have obtained liability insurance for clinical research. In case an unexpected serious health hazard occurs that is clearly related to this research, a compensation will be made according to the extent of the damage. Principal Investigators and Co-Investigators are required to have physician's liability insurance in case of liability.

# Saving records

This study is an invasive interventional study. As such, medical records will be appropriately kept for at least five years from the date that the study is reported complete, or at least 3 years since day of the final release of study results, whichever is later.

# Release of study results

　Before starting to enrol subjects in this study, the content of the study protocol will be registered in the public registration system, UMIN.

　We will present our results at academic conferences in Japan and abroad, and write an academic paper. When the final release of study results is complete, we will promptly register the hospital study plan and report to the hospital director.

# Study organisation

## Principal investigator

Representative of multicentre research

Department of Gastroenterology, Osaka City University Hospital

　Yasuaki Nagami 　　06-6645-2317、2318

## Participating facilities・Study responsible physicians in Osaka City University

Department of Gastroenterology, Osaka City University Hospital

Principal investigator：Yasuaki Nagami

Coordinator : Masafumi Yamamura

Investigators : Yasuhiro Fujiwara, Toshio Watanabe, Shusei Fukunaga, Masaki Ominami,

Hirotsugu Maruyama, Kojiro Tanoue, Kappei Hayashi, Yuki Kakiya, Taku Manabe

## Clinical trial office

Department of Gastroenterology, Osaka City University Hospital

Tel：06-6645-2316、2317

e-mail：[m2043767@med.osaka-cu.ac.jp](mailto:m2043767@med.osaka-cu.ac.jp), yasuaki-75@med.osaka-cu.ac.jp

Names of Researchers

## Data centre

Perform patient registration, assignment, and data management.

Data centre within the Osaka City University Hospital Center for Clinical Research and Innovation

TEL: 06-6645-3443

## Data and Safety Monitoring Committee

In order to objectively evaluate the safety of this study, (1) when a serious adverse event occurs, the Principal Investigator will report to the hospital director. He/she will also discuss with the Data and Safety Monitoring Committee whether or not the study should be continued. (2) In addition, since we assume the postprocedural bleeding rate to be 10–20%, we expect 10–20 postprocedural bleeding cases once we have 100 cases registered. Once we have over 10 cases of postprocedural bleeding, there will be a review by the Data and Safety Monitoring Committee. Furthermore, we will discuss the continuation of the trial by consulting with a member of the Clinical Study/Clinical Trial Advisory Committee.

Department of Gastroenterology, Osaka City University Hospital

Phone: 06-6645-2316, 2317

e-mail：m1265271@med.osaka-cu.ac.jp

Shuhei Hosomi

# Deviations or changes from the study protocol

If there is a significant deviation from the protocol, the Principal Investigator or the Co-Investigator will record the deviated item accompanied by an explanation.

# Monitoring/Quality Control of Data

Central monitoring will be performed by data centres for the purpose of improving the scientific and ethical quality of research and education. By confirming the requirements provided by the Principal Investigator, a monitor ensures that the clinical study is conducted properly, that necessary information is accurately recorded, and guarantees sufficient reliability of the data. We will not implement monitoring by visiting facilities.

Monitoring items

1. Study progress
2. Eligibility of registered cases
3. Reasons for protocol cancellation and termination
4. Protocol deviation
5. Serious adverse events
6. Adverse events
7. Other issues regarding study progress and safety

Monitoring Committee

Department of Gastroenterology, Osaka City University Hospital

Phone: 06-6645-2316, 2317

e-mail：m1265271@med.osaka-cu.ac.jp

Shuhei Hosomi

# Audit

The aim is to confirm that the study has been appropriately implemented, and to guarantee the reliability of data obtained. The Audit Committee may be established to visit each facility for an audit when it is deemed necessary by the Data and Safety Monitoring Committee and the Monitoring Committee members.

# References

1. 2012 Vital Statistics Monthly Annual Report: Ministry of Health, Labor and Welfare website<http://www.mhlw.go.jp>
2. Sawhney MS,Salfiti N,Nelson DB,Lederle FA, *et al.* Prevention of colorectal cancer by colonoscopic polypectomy. The National Polyp Study Workgroup. *N. Engl. J. Med.* 1993; 329: 1977-81
3. Magnus Løberg, Mette Kalager, Øyvind Holme, *et al.* Long-Term Colorectal-Cancer Mortality after Adenoma Removal. *N Engl J Med* 2014; 371:799-807
4. Hui AJ,Wrong RM,Ching JY,*et al*. Risk of colonoscopic polypectomy bleeding with anticoagulants and antiplatelet agents : analysis of 1657 cases.*Gastrointest. Endosc* . 2004;59:44-8
5. Garcia DA,Regan S,Henault LE,*et al*. Risk of thromboembolism with short-term interruption of warfarin therapy. *Arch Intern Med* 2008;168:63-9
6. Constans M, Stantamaria A, Mateo J *et al* .Low-molecular-weight heparin as bridging therapy during interruption of oral anticoagulation in patients undergoing colonoscopy or gastroscopy. *Int J Clin Pract* 2007;61:212-7.
7. James D.Douketis, Alex C. Spyropoulos, Scott Kaatz, *et al*. Perioperative Bridging Anticoagulation in Patients with Atrial Fibrillation. *N Engl J Med* 2015; 373: 823-833
8. David H, *et al.* Pacemaker or defibrillator surgery without interruption of anticoagularion.*N. Engl. J.* 2013; 368: 2084-93
9. Roberto T, Sant’anna, M.D, Tiago L, *et al*. Meta-Analysis of continuous oral anticoagulants versus heparin bridging in patients undergoing CIED surgery: reappraisal after the BRUISE study. *PACE* 2015; 38: 417–423
10. Nematullah A，et al．Dental surgery for patients on anticoagulant therapy with warfarin: a systemic review and meta-analysis．J Can Dent Assoc．2009 ; 75: 41-41i
11. 科学的根拠に基づく抗血栓療法患者の抜歯に関するガイドライン2010年版
12. Takuya Inoue,Tsutomu Nishida,Akira Maekawa, *et al*. Clinical features of post-polypectomy bleeding associated with heparin bridge therapy. *Digestive Endoscopy* 2014; 26: 243-249.
13. [Horiuchi A](http://www.ncbi.nlm.nih.gov/pubmed/?term=Horiuchi%20A%5BAuthor%5D&cauthor=true&cauthor_uid=24125514), [Nakayama Y](http://www.ncbi.nlm.nih.gov/pubmed/?term=Nakayama%20Y%5BAuthor%5D&cauthor=true&cauthor_uid=24125514), [Kajiyama M](http://www.ncbi.nlm.nih.gov/pubmed/?term=Kajiyama%20M%5BAuthor%5D&cauthor=true&cauthor_uid=24125514), *et al.* Removal of small colorectal polyps in anticoagulated patients: a prospective randomized comparison of cold snare and conventional polypectomy.　[*Gastrointest Endosc.*](http://www.ncbi.nlm.nih.gov/pubmed/24125514) 2014 Mar; 79 (3): 417-23.
14. 小林沙代, 他　抗凝固薬服用下の大腸ポリープ内視鏡的粘膜切除術、岡田雄介, 他　抗血栓薬服用者における内視鏡的大腸ポリープ切除術後出血の検討　第91回日本消化器内視鏡学会総会
15. Hideaki Harada et al. Endoscopic Submucosal Dissection With Continued Warfarin Compared With Heparin Bridge Therapy. GASTROINTESTINAL ENDOSCOPY Volume 83, No. 5S : 2016
16. Jonsson A, et al. [Kidney transplantation without interruption of warfarin.](http://www.ncbi.nlm.nih.gov/pubmed/26123573) Clin Transplant. 2015 Aug;29(8):665-6.
17. 偶発症に関する全国アンケート調査　2003年から2007年
18. 抗血栓薬服用者に対する消化器内視鏡診療ガイドライン　2012　日本消化器内視鏡学会
19. 大腸癌取扱い規約　第8版　大腸癌研究会

# Attached documents／Attached tables（as needed）
