# Supplementary material for: Continuous warfarin administration versus heparin bridging therapy in post colorectal polypectomy haemorrhage: a study protocol for a multicentre randomised controlled trial (WHICH study)
Source: Trials. 2021 Jan 7;22:33. doi: 10.1186/s13063-020-04975-y (PMC7791998; doi:10.1186/s13063-020-04975-y)
Supplement: Supplementary file 2 — Additional file 2. Statistical analysis plan. [file 13063_2020_4975_MOESM2_ESM.docx]

Statistical Analysis Plan

Continuous Warfarin administration versus Heparin bridging therapy in post colorectal polypectomy hemorrhage: A randomized controlled multicenter study

Short title: WHICH study

Principal investigator Yasuaki Nagami

Department of Gastroenterology, Osaka City University Graduate School of Medicine

Statistical Analysis Supervisor Hisako Yoshida

Department of Medical Statistics, Osaka City University Graduate School of Medicine

Version Number: 1.0 Date Created: April 1, 2020

Revision History

| Version Number | Date Created | Authors | Major changes |
| --- | --- | --- | --- |
| 1.0 | April 1, 2020 | YOSHIDA Hisako | Document created |

List of definition of terms and abbreviations

Terms used in this study protocol

| Term | Definition |
| --- | --- |
| Postoperative haemorrhage | Observation of any of the following within 28 days after surgery will be defined as postoperative haemorrhage.  -Bloody stool with an Hb decrease of 2 g/dL or more.  -Overt bloody stool treated with endoscopic haemostasis, angiography, surgery, and/or blood transfusion. |
| Case requiring haemostasis during surgery | A case in which haemostasis techniques such as clipping was performed where spontaneous haemostasis did not occur. |
| Warfarin therapeutic range | PT-INR of 1.5 or more and 3.0 or less  Refer to the Gastrointestinal Endoscopy Practice Guidelines for Patients on Antithrombotic Drugs by the Japanese Gastroenterological Endoscopy Society, 2012 |
| ESD | Endoscopic submucosal dissection;  A method in which a lesion is collectively removed by injecting sodium hyaluronate solution locally into the submucosal layer of the tumour and incising and peeling using an electric scalpel |
| Hybrid ESD | A method in which the submucosal layer is separated to perform a snare at the end after making a perilesional incision using an ESD knife or a snare tip |
| FAS | Full analysis set: Defined as subjects who have been assigned to this study, have taken the study drug at least once, and have been evaluated for efficacy at least once after drug administration. |
| PPS | Per protocol set: Target population that meets the protocol requirements. |

List of abbreviations for general items

| Abbreviation | Unabbreviated expressions (Japanese) |
| --- | --- |
| NBI | narrow band imaging (狭帯域光観察) |
| AFI | autofluorescence imaging (自家蛍光観察) |
| SSA/P | sessile serrated adenoma/polyp (大腸鋸歯状腺腫/ポリープ) |
| LST | **laterally spreading tumours (側方発育型腫瘍)** |
| CRF | case report form (症例調査票) |
| POD | postoperative day (術後日数) |
| IRB | Institutional Review Board (治験審査委員会) |
| DOAC | direct oral anticoagulant drugs (直接経口抗凝固薬) |
| ASA-PS | [American Society of Anaesthesiologists](https://en.wikipedia.org/wiki/American_Society_of_Anesthesiologists) - physical status classification (米国麻酔学会術前状態分類) |

Table of Contents

[Statistical Analysis Plan 1](#_Toc38107226)

[Definitions of Terms 3](#_Toc38107227)

[Abbreviations 3](#_Toc38107228)

[1. Scope of application 6](#_Toc38107229)

[2. Study purpose 6](#_Toc38107230)

[**2.1. Research hypothesis** 6](#_Toc38107231)

[3. Study design 6](#_Toc38107232)

[**3.1. Study design and plan** 6](#_Toc38107233)

[**3.2. Study population** 7](#_Toc38107234)

[3.2.1 Inclusion criteria 7](#_Toc38107235)

[3.2.2 Exclusion criteria 8](#_Toc38107236)

[**3.3. Randomisation** 8](#_Toc38107237)

[**3.4. Method of evaluation** 9](#_Toc38107238)

[(1) Patient background 9](#_Toc38107239)

[(2) The presence or absence of antiplatelet drug administration 9](#_Toc38107240)

[(3) Check for subjective symptoms and objective findings 10](#_Toc38107241)

[(4) Observation of lesions 10](#_Toc38107242)

[(5) Check for adverse events and side effects 10](#_Toc38107243)

[(6) The presence or absence of bloody stool 10](#_Toc38107244)

[(7) Haematology tests 10](#_Toc38107245)

[(8) Blood biochemistry tests 10](#_Toc38107246)

[(9) PT-INR 10](#_Toc38107247)

[(10) APTT 10](#_Toc38107248)

[4. Setting the number of subjects and the study period 11](#_Toc38107249)

[**4.1. Target number of subjects** 11](#_Toc38107250)

[**4.2. Study implementation period** 11](#_Toc38107251)

[5. Assessment parameters 12](#_Toc38107252)

[**5.1. Main assessment parameters** 12](#_Toc38107253)

[**5.2. Secondary assessment parameters** 12](#_Toc38107254)

[6. Target analysis population 12](#_Toc38107255)

[**6.1. Definition of target analysis population** 13](#_Toc38107256)

[**6.2. Definition of protocol deviations** 13](#_Toc38107257)

[7. Statistical analysis plan 14](#_Toc38107258)

[**7.1. Timing of analysis** 15](#_Toc38107259)

[**7.2. Target study group** 15](#_Toc38107260)

[7.2.1 Breakdown of subjects 15](#_Toc38107261)

[7.2.2 Characteristics of demographic and baseline values 16](#_Toc38107262)

[**7.3. Noninferiority analysis** 21](#_Toc38107263)

[7.3.1 Main assessment parameters 21](#_Toc38107264)

[7.3.2 Secondary assessment parameters (2), (3), (4), (5), (8), (10) 21](#_Toc38107265)

[**7.4. Superiority analysis** 22](#_Toc38107266)

[7.4.1 Secondary assessment parameters (1)(2) 22](#_Toc38107267)

[7.4.2 Secondary assessment parameters (3), (4), (5), (8), (10) 22](#_Toc38107268)

[7.4.4 Secondary assessment parameters (6) 22](#_Toc38107269)

[7.4.5 Secondary assessment parameters (7), (9) 23](#_Toc38107270)

# Scope of Application

This statistical analysis plan was prepared based on the study protocol "Continuous warfarin (Antithrombin therapy) administration versus heparin bridge therapy in post colon polypectomy haemorrhage" (WHICH study), 6th Edition (created August 31, 2018). It describes the statistical methodologies and details of the statistical analysis methods to be applied in the study protocol.

# Study Purpose

Compared with conventional method where patients on warfarin potassium (warfarin) switch to heparin sodium (heparin) for endoscopic colorectal polypectomy, this study was designed to demonstrate that the trial method of endoscopic colorectal polypectomy under continuous warfarin is not inferior to the conventional heparin bridge method in terms of the frequency of postoperative bleeding events.

## **2.1. Research Hypothesis**

Colorectal polypectomy with continued anticoagulants may have a lower bleeding rate than heparin-bridged cases, and is not inferior to heparin-bridged cases.

# Study Design

## **3.1. Study Design and Plan**

In this study, patients on warfarin with colorectal polyp, the target disease, who are scheduled for colorectal polypectomy are divided into standard treatment group (heparin bridge group) and trial treatment group (continued warfarin group) before the procedure. The postoperative haemorrhage rate after endoscopic colorectal polypectomy is compared. This is a prospective, multicentre, dynamically randomised, two-group parallel non-inferiority trial.

## Scheme


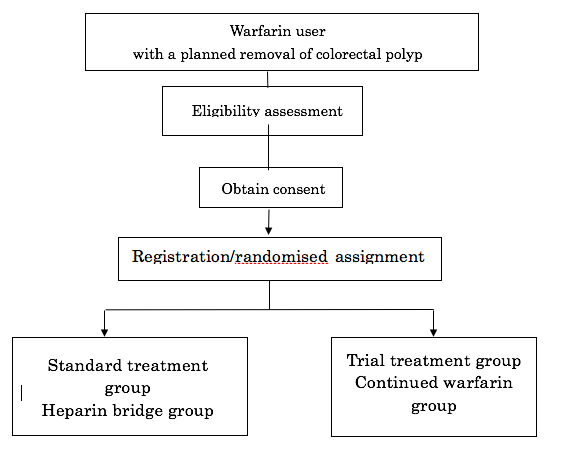


## **3.2. Study Population**

　Patients who underwent consultation at institutions participating in this study, who are taking warfarin in an outpatient or inpatient setting, and who are scheduled to undergo colorectal polypectomy.

## 3.2.1 Inclusion Criteria

Patients who satisfy the following criteria are selected.

1. Patients with polyps that can undergo en-bloc resection of the large intestine (cecum, colon, and rectum), and who are scheduled for endoscopic colorectal polypectomy
2. Patients who have been taking warfarin for at least 2 weeks prior to endoscopic colorectal polypectomy
3. Patient is at least 20 years old at the time of obtaining consent
4. Written consent is provided based on the patient’s free will, after he or she has a thorough understanding of the explanation given regarding study participation

[Criteria rationale]

1) 2) Target patients who undergo endoscopic treatment while continuing to take anticoagulants.

3) 4) For safety considerations

Note 1) Patients on DOAC are excluded from this study since the rate of postoperative bleeding after endoscopic colorectal polypectomy has not been clarified.

Note 2) Included also are cases that are diagnosed as resectable en-bloc, but actually undergo piecemeal resection.

### 3.2.2 Exclusion Criteria

Patients who meet any of the following criteria are excluded.

1. Patients with a history of enrolment in this study
2. Patients with inflammatory bowel disease, familial adenomatous polyposis, and Peutz-Jeghers syndrome
3. Cases whose clinical course cannot be followed up to 28 days after treatment
4. Any case with a history of bleeding with blood transfusion of 2 RBC units or more, Hb reduction of ≧ 2g/dL or haemostasis treatment within 6 weeks before surgery
5. Dialysis patients
6. Patients whose blood test show a platelet count of less than 50,000/μL within 12 weeks before surgery
7. Patients with coagulation dysfunctions
8. Pregnant patients
9. Lactating patients
10. Patient is allergic to heparin and warfarin
11. Other cases determined to be unfit for study by a head doctor or doctor in charge

[Criteria rationale]

1)~4) Due to their impact on effectiveness evaluation

5)~11) For safety considerations

## **3.3. Randomisation**

In this study, cases will undergo dynamic randomisation using the online registration allocation system at the data centre within the Osaka City University Hospital Center for Clinical Research and Innovation. The following three allocation adjustment factors will be used: (1) facilities, (2) the number of known lesions, and (3) concomitant use of antiplatelet drugs. For patients who provide written consent after receiving study explanations, we will confirm that they satisfy the inclusion criteria and that they do not meet any of the exclusion criteria.

## **3.4. Method of evaluation**

　The following information will be collected according to the schedule.

1. Patient background

Medical record number (ID number), sex, date of birth (age), surgical history, oral dose of warfarin, disease names for which anticoagulants are prescribed (atrial fibrillation, venous thrombosis, myocardial infarction, pulmonary embolism, cerebral embolism, coronary artery bypass surgery, artificial valve replacement surgery, and others)

Comorbidity (diabetes, hypertension, heart disease, respiratory disease, renal disease, liver disease, brain disease, endocrine/metabolic disease, autoimmune disease, malignancy, and others)

ASA-PS

Note 1) In facilities where reporting personal information (ID and date of birth) is restricted, the facility's patient identification code and the age of the patient at the time of consent will be recorded. This information will be stored by patient identification code at each facility.

Note 2) ASA-PS ([American Society of Anesthesiologists](https://en.wikipedia.org/wiki/American_Society_of_Anesthesiologists) - Physical status classification)

　　Class 1: Healthy patients (except conditions requiring surgery) (inguinal hernia, uterine fibroids, appendicitis surgery, among others)

　　Class 2: Patients with mild to moderate systemic disease

　　　Mild diabetes, mild essential hypertension, anaemia, neonates and those who are 80 years or older, severe obesity, chronic bronchitis, other tumours, etc.

Class 3: Patients with severe systemic disease

Severe diabetes mellitus, moderate/advanced lung disease (COPD), controlled ischaemic heart disease (history of PCI/CABG), cerebral infarction, liver cirrhosis (Child-Pugh class B/C), dialysis, multiple malignancy metastases, etc.

Class 4: Patients with severe life-threatening systemic diseases

Multiple organ failure, and others.
Class 5: Near-death patients who cannot survive without surgery

Shock due to myocardial infarction, ruptured aortic aneurysm, severe pulmonary embolism, etc.
Class 6: Brain dead patients

1. The presence or absence of antiplatelet drug administration

If a patient was on an antiplatelet drug prior to trial drug administration, the Principal Investigator will note the drug name, the presence or absence of drug withdrawal and its duration, reason for antiplatelet drug use (thromboembolism high risk group/low risk group), and whether or not the drug was replaced on the CRF.

1. Check for subjective symptoms and objective findings

Confirm by patient interview. If available, a symptom diary may be referred.

1. Observation of lesions

Deepest insertion point, the presence or absence of colorectal surgery, number of lesions, lesion number, lesion size, macroscopic type, tissue type, lesion site, the presence or absence of immediate postsurgical bleeding, treatment method (polypectomy/EMR), the type of local injection solution, number of clippings performed after resection, whether or not the lesion was collected, snare type (bipolar/monopolar), the presence or absence of immediate postsurgical haemorrhage without spontaneous haemostasis, the presence or absence of perforation during treatment, treating physician's years of experience

Note) Histological type will be confirmed during the outpatient visit after discharge

1. Check for adverse events and side effects
2. The presence or absence of bloody stool
3. Haematology test

Peripheral blood count: white blood cells, red blood cells, haemoglobin, haematocrit, and platelets

1. Blood biochemistry test

Renal function: BUN, Cre

Liver function: AST, ALT, T-bil

Electrolytes: Na, K, Cl,

Inflammatory reaction: CRP

1. PT-INR
2. APTT

In case of postsurgical bleeding, re-examine (7), (8), (9), and (10). The measurement of APTT with postoperative bleeding is not required in the trial treatment group.

**Table 3-1 Schedule**

| Period | Outpatient | 5~1 day prior to admission date | 4 to 1 day before planned treatment date | Treatment date | The day after treatment |  | Postoperative bleeding | Postoperative day 28 |
| --- | --- | --- | --- | --- | --- | --- | --- | --- |
| Patient visit | Visit１ | Admission | Admission | Admission | Admission | Admission →  Discharge | Emergency visit | Visit 2 |
| Obtain consent | ○ | |  |  |  |  |  |  |
| Patient background check | ○ | ● |  |  |  |  |  |  |
| Subjective symptoms/Objective findings | ○ | ● |  | ○ | ○ | ○ | ○ |  |
| Examination  (Physical findings/ Observe adverse events) | ○ | ● |  | ○ | ○ | ○ | ○ |  |
| Antiplatelet drug administration check | ○ | ● |  |  |  |  |  |  |
| Adverse event and side effect check |  |  | ● | ● | ● |  |  |  |
| Presence or absence of bloody stool |  |  |  |  | ○ | ○ | ○ |  |
| Warfarin | ○ | ○ | Discontinue in heparin bridge group Continue in warfarin continued group | | ○ | ○ | ○ |  |
| Heparin bridge |  |  |  |  |  |  |  |  |
| Endoscopy exam |  |  |  | ○ |  |  | ○ |  |
| Lesion observation |  |  |  | ○ |  |  |  |  |
| Haematology test | ○ |  |  |  | ○ |  | ○ | ○ |
| Biochemistry test | ○ |  |  |  | ○ |  | ○ |  |
| PT－INR | ○ | ○ | ● | ○ | ○ | ● | ○ | ○ |
| APTT | ○ | ● | ● | ● | ● | ● | ● |  |

○ indicates items that must be implemented; ● indicates items that will be implemented as necessary

Note) Consent can be obtained in the outpatient setting as well. In addition, a patient can be treated if the PT-INR becomes 3 or less after hospitalisation.

Note) If a blood test cannot be done on postoperative day 28, it can be done up to postoperative day 35.

#

# Setting the number of study subjects and the study period

## **4.1. Target number of subjects**

　Target number of cases: 316 cases; Osaka City University Hospital: 90 cases

## Rationale

　　　Few reports have examined the post-procedural bleeding rate after endoscopic polypectomy among patients who continued warfarin. According to a report by Horiuchi et al.^12)^, the rate of postoperative bleeding was 14% among those who continued warfarin, and 20% in patients who switched from warfarin to heparin. Based on these findings, the bleeding rate in this study can be assumed to be similar. We assume the post-procedural bleeding rate of 14% for warfarin continued cases, and 20% for heparin bridged cases. Non-inferiority margin was set to 5%. Given α value of 0.05 and power of 0.8, we considered an enrolment of about 144 cases in each group to be appropriate. The target number of cases was set at 158 in each group, for a total of 316, assuming that a little less than 10% of cases may be discontinued or are ineligible.

## **4.2. Study implementation period**

Case registration period: From the date of approval to August 31, 2022 (registration deadline August 31, 2022)

Total research period: From the date of approval to August 31, 2023

Registration period: 6 years.

Follow-up period: Up to 28 days after surgery.

Total research period: 7 years

# Assessment parameters

## **5.1.** Primary assessment parameters

　Postoperative bleeding rate　Cases with postoperative bleeding/cases that underwent polypectomy

[Criteria rationale]

If the rate of postoperative bleeding with continued warfarin is not inferior to that of heparin bridge, endoscopic colorectal polypectomy with continuous warfarin could become the standard treatment. This leads to reduced burden associated with the heparin bridge on those involved in care and patients, and reduced cost.

Postoperative bleeding　When bleeding is observed with one or more of the following within 28 days after surgery

-Bloody stool with an Hb decrease of 2 g/dL or more.

- Overt bloody stool treated with endoscopic haemostasis, angiography, surgery, and/or blood transfusion.

Note) Even if there is no active bleeding and the source of bleeding is unknown during urgent endoscopy, haemostasis techniques (additional clipping) will be performed if haemorrhage is strongly suspected (adhesion of blood clots or exposed blood vessels).

Emergency endoscopy criteria

Emergency lower gastrointestinal endoscopy will be performed in the following cases: twice or more of persistent bloody stool without a sign of improvement; bloody stool with changes in vital signs (systolic blood pressure <100 mmHg or pulse >90 beats/min); or bloody stool with an Hb decrease of 2 g/dL or more.

## **5.2.** Secondary assessment parameters:

1. Cumulative bleeding rate
2. Rate of overt haemorrhage that does not satisfy the definition of haemorrhage after endoscopic polypectomy
3. Incidence rate of haemorrhage that required haemostasis during endoscopic polypectomy

Cases that required haemostasis during surgery: Cases where a haemostasis technique such as clipping was performed for bleeding that did not undergo spontaneous haemostasis.

1. Intraoperative bleeding during endoscopic colorectal polypectomy requiring angiography, surgery and/or blood transfusion
2. Total bleeding rate (postoperative bleeding + ② + ③)
3. Risk factors for postoperative bleeding
4. Number of hospitalisation days
5. Incidence rate of thromboembolism
6. PT-INR 28 days after surgery (If it is difficult to perform a blood test on postoperative day 28, it can be performed up to postoperative day 35).
7. Percentage of serious adverse events

[Criteria rationale]

- - 1. To clarify whether there is a difference in the postprocedural bleeding rate, as well as timing of bleeding, between warfarin continued cases and heparin-bridged cases.
    2. For bleeding that did not require emergency treatment, we aim to clarify whether there is a difference in bleeding rate between warfarin continued cases and heparin bridged cases.
    3. With regard to intraoperative bleeding, we aim to clarify whether there is a difference in bleeding rate between warfarin continued cases and heparin-bridged cases.
    4. To clarify whether severe intraoperative bleeding that requires aforementioned treatment can occur in warfarin continued and heparin replaced cases, and if so, whether there is a difference in bleeding rate.
    5. All treatment-related bleeding will be assessed to clarify the difference between the warfarin continued group and the heparin-bridged group.
    6. This study is a non-inferiority study. However, we also aim to clarify whether warfarin administration or heparin bridge may be a risk factor for postoperative haemorrhage, and whether there are other risk factors for postoperative haemorrhage.
    7. Heparin-bridged patients are expected to have a longer hospital stay than those who continued to take warfarin.
    8. To clarify whether the incidence of thromboembolism in heparin-bridged patients differs from that in warfarin continued patients.
    9. To clarify whether PT-INR returns to preprocedural levels within 28 days in heparin bridged group, since it may affect the incidence of thromboembolism and postoperative bleeding.
    10. Because the lower the incidence of serious adverse events between the two groups, the better.

# Target analysis population

## **6.1. Definition of target analysis population**

The breakdown of the study’s subjects is outlined below.

1. Example of consented cases

　　Defined as a subject who is confirmed to have provided written consent.

1. Example of randomisation

　　Cases that were randomly assigned among the consented cases

1. Target population of the safety analysis

　　Defined as a group that satisfies the following criteria among consented cases. However, cases with no primary assessment parameter are excluded.

1. Randomised
2. No withdrawal of consent regarding provision of information
3. Target population of effectiveness analysis
4. Full Analysis Set (FAS)

Defined as a group that meets the following criteria among consented cases. However, cases with no primary assessment parameter are excluded.

- - 1. Randomly assigned
    2. No withdrawal of consent regarding provision of information
    3. Not found to be ineligible after randomisation

1. Per Protocol Set (PPS)

Defined as a group that does not largely deviate from the protocol among the full analysis set (FAS).

## **6.2. Definition of protocol deviations**

If any of the following criteria are met, the protocol treatment will be discontinued.

1. Cases with PT-INR >3 (on study day): However, if the treatment is postponed and the treatment is performed once the PT-INR is 3 or less, it will not be considered a protocol deviation.
2. Cases of ESD and Hybrid ESD
3. Cases where endoscopic colorectal polypectomy is cancelled before completion of the procedure (the procedure will be deemed complete with the constriction of a snare)
4. If a gastrointestinal perforation is seen during or after endoscopic colorectal polypectomy
5. When an additional surgery is performed within 28 days after endoscopic colorectal polypectomy
6. When a subject requests to leave the study or withdraws consent
7. Other reasons given by a subject (too busy, transfer to another hospital, relocation, etc.)
8. If a subject is found to not satisfy the eligibility criteria after registration
9. When continuing the study is difficult due to worsening complications
10. When continuing the study is difficult due to adverse events
11. If a physician decides that cancelling the study is appropriate for any other reasons

Note) Hybrid ESD: A technique using an ESD-specific knife or a snare tip. After making an incision around the lesion, the submucosal layer will be separated to complete snaring

# Statistical analysis plan

Below, unless otherwise specified, data will be aggregated for each group (standard treatment group and trial treatment group). In addition, unless otherwise specified, a ratio is calculated with the aggregated target group size as the denominator.

For categorical data and quantitative data, the descriptive statistics shown in Table 7-1 will be calculated. The number of digits displayed in each statistic will be in accordance with Table 7-2. Table 7-3 includes the definition of terms used in this chapter.

Missing values are not considered in the main analysis and the secondary analysis. However, for sensitivity analysis, missing value complementation such as the multiple substitution method will be performed as appropriate, in order to evaluate the stability of analysis results.

R (version 3.6.1 or later) is used for statistical analysis. Reported p-values will be two-sided, but the p-value for noninferiority evaluation will be one-sided.

#### Table 7-1 Descriptive statistics

| Item | Statistics to calculate | |
| --- | --- | --- |
| Categorical data | Frequency | Number of subjects, ratio |
|  | Cross frequency | Number of subjects, ratio |
| Continuous data | Statistic | Number of subjects, arithmetic mean, standard deviation, median, minimum value, maximum value |

#### Table 7-2 Number of statistical digits displayed

| Item | Numerical display |
| --- | --- |
| Arithmetic mean, median | Round the second significant digit and display data with one significant figure |
| Standard deviation | Round the third significant digit and display data with two significant figures |
| Minimum value, maximum value | Display the same number of significant digits as the data |
| Number of Subjects | Display as an integer |
| P-value | Round the fifth decimal place to display data with 4 decimal places.  However, if the value is less than 0.0001, display as <0.0001. |

#### Table 7-3 Definition of terms

| Term | Definition |
| --- | --- |
| Age (years) | Age on the day of consent |
| BMI | Weight (kg)/(height (cm)/100)^2^ |

BMI, body mass index

## **7.1. Timing of analysis**

　This analysis will be carried out when the investigation, observation, and examination of all subjects are complete and data is fixed. However, the main assessment parameter analysis and the quality control process may be ongoing given the need for planning and preparation of publications and conference presentations. If an additional analysis is deemed necessary, this analysis plan will be revised for further implementation.

## **7.2. Target study group**

### 7.2.1 Breakdown of subjects

　The subject details are summarised by group in a CONSORT diagram. The definition and breakdown of the group that correspond to the diagram "box" are as follows.

1. Cases in which consent was confirmed
2. Cases that were randomly assigned

The breakdown of unassigned cases will be shown.

1. FAS

Cases included in (2) excluding the following cases: ineligible cases, cases where consent regarding data use was withdrawn, cases where endoscopic resection was not completed, and cases without any subsequent information.

1. Safety analysis group

Follows (3); it will be included as a breakdown in (2).

1. PPS

Follows (3); it will be included as a breakdown in (2).

(6) Tracking status

The follow-up status for each group is summarised as follows:

Death during the study period

Cases where follow-up was discontinued due to causes besides death

Untraceable cases due to hospital transfer/relocation

Untraceable cases due to reasons other than the above

Cases that were followed until the end of the study

### 7.2.2 Characteristics of demographic and baseline values

Target of analysis: Consented cases/assigned cases, FAS

Items for analysis: Each item in Table 7-4

Analysis method: Each item for the analyses described above will be aggregated and analysed per group. The clinical laboratory values of insured medical treatment will be tabulated and analysed before the beginning of study and during secondary registration/allocation. To examine the balance between the two groups, the Chi-squared test will be performed for categorical data, and T-test will be performed for continuous data. If distribution symmetry/normality is maintained by logarithmic transformation of clinical laboratory values, summary statistics will be calculated after logarithmic transformation. In such cases, aforementioned intergroup tests will also be done after logarithmic transformation (whether or not the data should be transformed will be determined by a statistician through a blind review along with the group). The baseline medication will be categorised by main drug indication groups, and summarised by group. The comparison between groups will be performed by the Chi-squared test.

1) Frequency summary of categorical data and descriptive statistics of quantitative data

#### Table 7-4 Summary items

| Item | Categorisation: |
| --- | --- |
| Age (years) | (Calculate descriptive statistics) |
|  | Under 65, 65 years and above |
| Sex | Male, Female |
| Number of polyps known in advance | (Calculate descriptive statistics) |
| Concurrent use of antiplatelet drugs | No, Yes |
| Surgical history | No, Yes |
| Oral warfarin dose (mg/day) | (Calculate descriptive statistics) |
| Disease name for which anticoagulant is prescribed | Atrial fibrillation, venous thrombosis, myocardial infarction, pulmonary embolism, cerebral embolism, coronary artery bypass surgery, artificial valve replacement, etc. |
| Diabetes | No, Yes |
| Hypertension | No, Yes |
| Heart disease | No, Yes |
| Respiratory disease | No, Yes |
| Renal disease | No, Yes |
| Liver disease | No, Yes |
| Brain disease | No, Yes |
| Endocrine/metabolic diseases | No, Yes |
| Autoimmune disease | No, Yes |
| Malignancy | No, Yes |
| Malignancy (disease name) | Free entry |
| Other | No, Yes |
| ASA-PS | Class 1, Class 2, Class 3 |
| CHADS2 score | 0 point, 1 point, 2 points, 3 points |
| Concurrent use of antiplatelet drug | No |
| Antiplatelet drug 1 (generic name) | Free entry |
| Antiplatelet drug 2 (generic name) | Free entry |
| Antiplatelet drug 3 (generic name) | Free entry |
| Reason for use | Free entry |
| Discontinuation of drug | No, Yes |
| Drug holiday | Drug withdrawal start date and end date |
| Drug replacement | No, Yes |
| Replaced drug name | Free entry |
| WBC (/μL) | (Calculate descriptive statistics) |
| RBC (x 10,000/μL) | (Calculate descriptive statistics) |
| Hb (g/dL) | (Calculate descriptive statistics) |
| Ht (%) | (Calculate descriptive statistics) |
| Platelet count (x10,000/μL) | (Calculate descriptive statistics) |
| BUN (mg/gL) | (Calculate descriptive statistics) |
| Cre (mg/dL) | (Calculate descriptive statistics) |
| AST (IU/L) | (Calculate descriptive statistics) |
| ALT (IU/L) | (Calculate descriptive statistics) |
| T-Bill (mg/dL) | (Calculate descriptive statistics) |
| Na (mEq/L) | (Calculate descriptive statistics) |
| K（mEq/L） | (Calculate descriptive statistics) |
| Cl（mEq/L） | (Calculate descriptive statistics) |
| CRP（mg/dL） | (Calculate descriptive statistics) |
| PT-INR | (Calculate descriptive statistics) |
| APTT（sec） | (Calculate descriptive statistics) |
| Final heparin dose (unit) | (Calculate descriptive statistics) |
| Heparin administration period (days) | (Calculate descriptive statistics) |
| Warfarin discontinuation period (days) | (Calculate descriptive statistics) |
| Patient discharge before PT-INR was within therapeutic range | No, Yes |
| Endoscopic polypectomy cancellation/postponement | No postponement, postponement, cancellation |
| Reason for polypectomy cancellation/postponement | Free entry |
| Years of experience of treating physician (years) | (Calculate descriptive statistics) |
| Deepest point of insertion | Cecum, ascending colon, transverse colon,  Sigmoid colon, rectum |
| Surgery of large intestine | No, Yes |
| Type of surgical procedure for large intestine | Free entry |
| Snare type | Bipolar, monopolar |
| Blood transfusion | No, Yes |
| Blood transfusion volume (unit) | (Calculate descriptive statistics) |
| Lesion site | Free entry |
| Lesion size (mm) | (Calculate descriptive statistics) |
| Macroscopic type | Is (sessile), Isp (subpedunculated), Ip (pedunculated), IIa (superficial elevated), IIb (superficial flat), IIc (superficial depressed),  IIa + dep, Is + IIa, IIa + Is, Is + IIc, IIc + Is, IIa + IIc, IIc + IIa, etc. |
| Macroscopic type (other details) | Free entry |
| Histologic type | Not collected,  Hyperplastic polyp  (Inflammatory polyp),  Adenoma (serrated adenoma),  SSA/P  Lesions including cancer (Tub)  Lesions including cancer (Tub in adenoma)  Lesions including cancer (Tub with adenoma) Lesion with cancer (other) |
| Histologic type (other details) | Free entry |
| Method of Treatment | Polypectomy, EMR (mucosal resection) |
| Type of local injection solution | Normal saline, glucose solution, glyceol, hyaluronic acid, mixed solution |
| Mixture details (local injection solution/mixing ratio) | Free entry |
| Clipping performed after polypectomy | No, Yes |
| Reason for no prophylactic clipping | Piecemeal removal, procedural difficulty due to large size, other |
| Reason (other details) | Free entry |
| Perforation during treatment | None, Yes (Reefing possible with an endoscope) |
| Perforation during treatment | Conservative treatment with caution, surgery required |
| Perforation (surgical procedure) | Free entry |
| Bleeding without spontaneous haemostasis immediately after treatment | No, Yes |
| Endoscopic haemostasis | No, Yes |
| Endoscopic haemostasis technique | Clipping, haemostatic coagulation device, etc. |
| Haemostatic technique (number of clippings) | (Calculate descriptive statistics) |
| Haemostasis technique (haemostatic coagulation device) | Free entry |
| Haemostasis difficulty with a haemostatic technique | No, Yes |
| Angiography | No, Yes |
| Surgery | No, Yes |
| Name of adverse event | Free entry |
| Adverse event content | Bloody stool, thromboembolism, etc. |
| Adverse event outcome | Recovery, remission, unrecovered, presence of sequela, death, unknown |
| Serious adverse events | Non-serious, serious |
| Re-bleeding after haemostasis | First time bleeding, re-bleeding |
| Systolic blood pressure (mmHg) | (Calculate descriptive statistics) |
| Diastolic blood pressure (mmHg) | (Calculate descriptive statistics) |
| Pulse (/min) | (Calculate descriptive statistics) |
| Postoperative bleed | No, Yes |
| Postoperative bleed detail | -Bloody stool with a Hb decrease of 2 g/dL or more  -Patient has an overt bloody stool and received treatment including endoscopic haemostasis, angiography, surgery, and blood transfusion |
| Heparin administration | No, Yes |
| Heparin discontinuation | No, Yes |
| Warfarin discontinuation | No, Yes |
| Antiplatelet drug 1 discontinuation | No, Yes |
| Antiplatelet drug 2 discontinuation | No, Yes |
| Antiplatelet drug 3 discontinuation | No, Yes |
| Emergency endoscopy | No, Yes |
| Cancellation | Yes |
| Reason for cancellation | Free entry |

## **7.3.** **Non-inferiority analysis**

### 7.3.1 Main assessment parameters

(1) Main analysis

　Analysis target: FAS, PPS, assigned cases (FAS will be the main analysis)

　Analysis method: The following analyses will be performed on the parameters above.

- - - 1. The statistical significance of the event incidence ratio between groups is evaluated, using postoperative bleeding as the outcome. We test the null hypothesis that the risk ratios of both groups are equal (= 1), using a generalised linear model with groups as fixed effects and allocation adjustment factor as a covariate. The three allocation adjustment factors are facilities, the number of lesions known in advance, and the antiplatelet drug. The significance level is 5% (one-sided), and the 95% confidence interval will be calculated. The trial treatment will be considered noninferior to standard treatment if the risk ratio's 95% confidence interval does not exceed 1.05.
      2. Analysis that complements the main analysis

　Analysis target: FAS, PPS, allocated cases (FAS will be the main analysis)

　Analysis method: The following analyses will be performed on the parameters above. Deaths, fatal events, and untraceable cases other than those included in the main assessment parameters will be treated as cancelled.

1. A Kaplan-Meier curve of the cumulative event incidence rate for each group will be created, and its 95% confidence interval (after double logarithmic transformation) will be calculated. Using the Cox regression, the point estimate of the group effect hazard ratio and its 95% confidence interval will be calculated. A forest plot of hazard ratios and confidence intervals will be created for the main assessment parameters and each event.

### 7.3.2 Secondary assessment parameters (2), (3), (4), (5), (8), (10)

Analysis target: FAS, PPS, allocated cases (FAS will be the main analysis)

Analysis method: The following analyses will be performed on the parameters above.

1. The statistical significance of the event incidence ratio between groups is evaluated with the event of each assessment parameter as the outcome. We test the null hypothesis that the risk ratios of both groups are equal (=1), using a generalised linear model with groups as fixed effects and allocation adjustment factor as a covariate. The three allocation adjustment factors are facilities, the number of lesions known in advance, and the antiplatelet drug. The significance level is 5% (one-sided), and the 95% confidence interval will be calculated. The trial treatment will be considered noninferior to standard treatment if the risk ratio's 95% confidence interval does not exceed 1.05.

## **7.4. Superiority analysis**

### 7.4.1 Secondary assessment parameters (1)(2)

The following analyses are performed for the secondary assessment parameters (1) and (2). Deaths and fatal events other than those included in secondary assessment parameters (1) and untraceable cases are treated as cancelled.

1. A Kaplan-Meier curve of cumulative incidence for each group will be created and its 95% confidence interval will be calculated. The confidence interval will be calculated for the double logarithmic transformation value.
2. The log rank test will be performed for comparison between groups (superiority).

### 7.4.2 Secondary assessment parameters (3), (4), (5), (8), (10)

The following analyses are performed on the title assessment parameters. Deaths and fatal events other than those included in each assessment parameters and untraceable cases are treated as cancelled.

1. A Kaplan-Meier curve of cumulative incidence for each group will be created and its 95% confidence interval will be calculated. The confidence interval will be calculated for the double logarithmic transformation value.
2. The log rank test will be performed for comparison between groups (superiority).

### 7.4.4 Secondary assessment parameters (6)

The following analyses are performed on the title assessment parameters. Deaths and fatal events other than those included in each assessment parameters and untraceable cases are treated as cancelled.

1. In order to identify risk factors, we use the Cox regression model with the allocation adjustment factor and other covariates to find the point estimate of the group effect hazard ratio and its 95% confidence interval. For the main assessment parameters, a forest plot of hazard ratios and confidence intervals for each variable will be created.
2. The risk factor candidates are listed in Table 7.4. The number of factors used in the model will be determined by the statistician according to the number of events.

### 7.4.5 Secondary assessment parameters (7), (9)

The following analyses are performed on the title assessment parameters.

1. For each assessment parameter, the statistical significance of the difference between assigned groups will be evaluated. Multiple regression analysis is performed with each assessment parameter as the outcome, the assigned group as the explanatory variable, and the stratification factor as a covariate.
2. The number of hospitalisation days is defined as the number of days from the treatment date to the discharge date (the treatment date and discharge date are also each counted as one day).
